# Supplementary material for: Optimizing cross-domain transfer for universal machine learning interatomic potentials
Source: Nat Commun. 2026 Mar 3;17:3432. doi: 10.1038/s41467-026-70195-8 (PMC13076606; doi:10.1038/s41467-026-70195-8)
Supplement: Supplementary file 1 — Supplementary information: Optimizing Cross-Domain Transfer for Universal Machine Learning Interatomic Potentials [file 41467_2026_70195_MOESM1_ESM.pdf]

Supplementary information: Optimizing Cross-Domain Transfer for Universal Machine Learning Interatomic Potentials

Jaesun Kim[1†], Jinmu You[1†], Yutack Park[1], Yunsung Lim[2], Yujin Kang[1], Jisu Kim[1], Haekwan Jeon[1], Suyeon Ju[1], Deokgi Hong[1], Seung Yul Lee[3], Saerom Choi[1,4], Yongdeok Kim[4], Jae W. Lee[3], Seungwu Han[1,2,5*]

[1]Department of Materials Science and Engineering, Seoul National University, Seoul, 08826, Republic of Korea.
[2]Research Institute of Advanced Materials, Seoul National University, Seoul, 08826, Republic of Korea.
[3]Department of Computer Science and Engineering, Seoul National University, Seoul, 08826, Republic of Korea.
[4]AI Center, Samsung Electronics, Suwon, 16678, Republic of Korea.
[5]Center for AI and Natural Sciences, Korea Institute of Advanced Study, Seoul, 02455, Republic of Korea.

*Corresponding author(s). E-mail(s): hansw@snu.ac.kr;
†These authors contributed equally to this work.

Supplementary Table 1: Composition of the domain-bridging database. The fraction represents the sampling ratio relative to the original database.

Database	Number of samples	Fraction (%)
MatPES	300	0.07
OC20	18,000	0.06
OC22	4,500	0.05
ODAC23	2,400	0.06
OMOL25	74,100	0.12
QCML	25,200	0.14

Supplementary Table 2: Statistics of molecule-containing structures in crystal database. Structures were sampled at 1% from each original training database. The ratio indicates the fraction of molecule-containing structures among the sampled data. Molecule-containing structures were identified using two criteria: (i) the presence of at least one H, C, N, O, or F atom, and (ii) the existence of two or more disconnected clusters in a graph representation of the structure. The graph was constructed using covalent-radius-based cutoffs as implemented in ASE, with a neighbor-list skin distance of 0.3 Å for robustness [1, 2]. Edge between two atoms is created if the interatomic distance is less than 1.3×(sum of covalent radii). The same analysis was also applied to a 2×2×2 supercell to correctly handle single-molecule-in-vacuum configurations under periodic boundary conditions.

Database	Number of sampled structures	Number of molecule-containing structures	Fraction (%)
MPtrj	15,802	1,488	9.42
Alex	120,335	4,186	3.48
OMat24	1,015,901	9,370	0.92

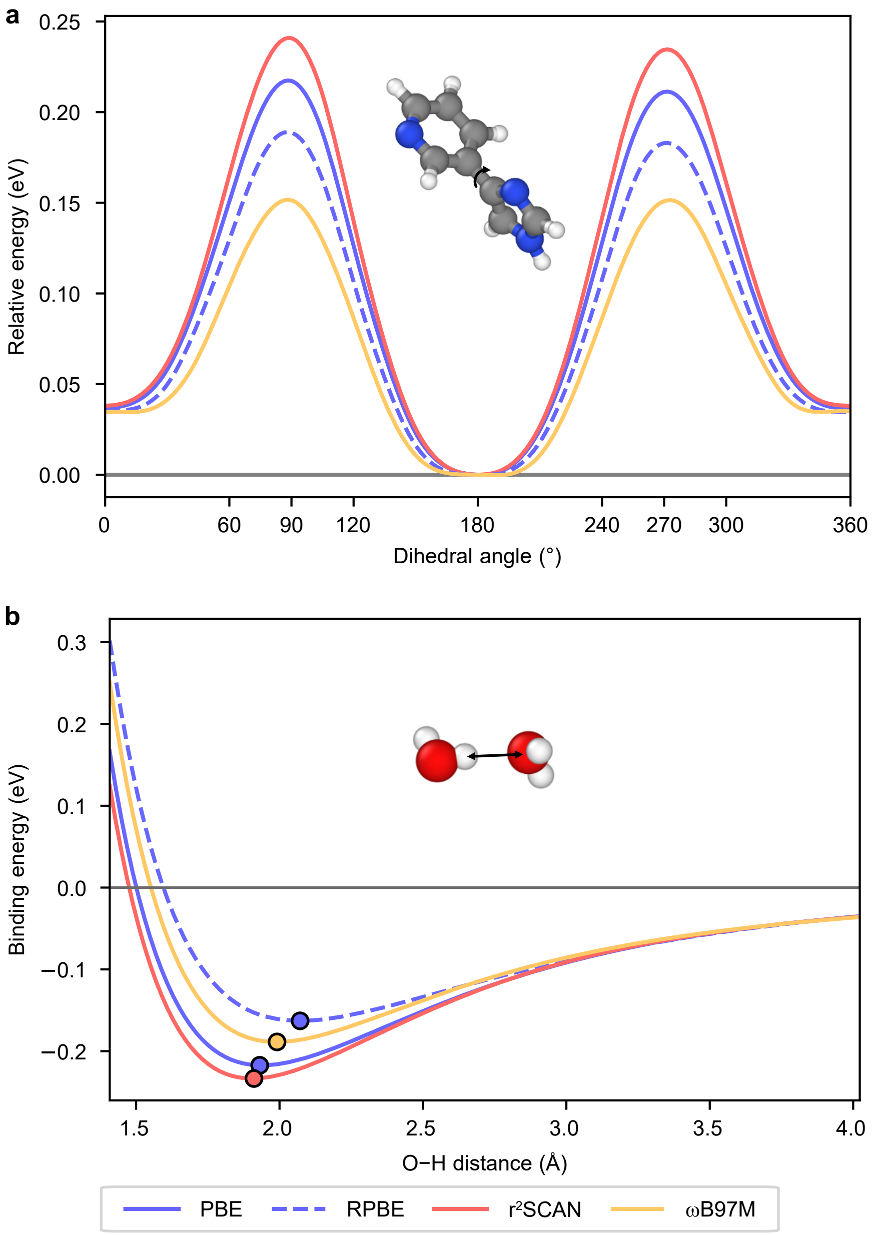

Supplementary Figure 1: **Potential energy surfaces of molecular systems with varying ab initio method.** Blue lines indicate PES obtained by GGA-type functionals, while red and yellow lines illustrate PES calculated with r^2SCAN and ωB97M functional, respectively. Among blue lines, solid lines and dashed lines corresponds to PBE and RPBE PES. **a** Torsional PES obtained by rotating dihedral angle of a biaryl molecule. **b** Energy landscape of water molecule dimer by varying distance between the two. Circle markers indicate equilibrium distance with the lowest energy for each functional.

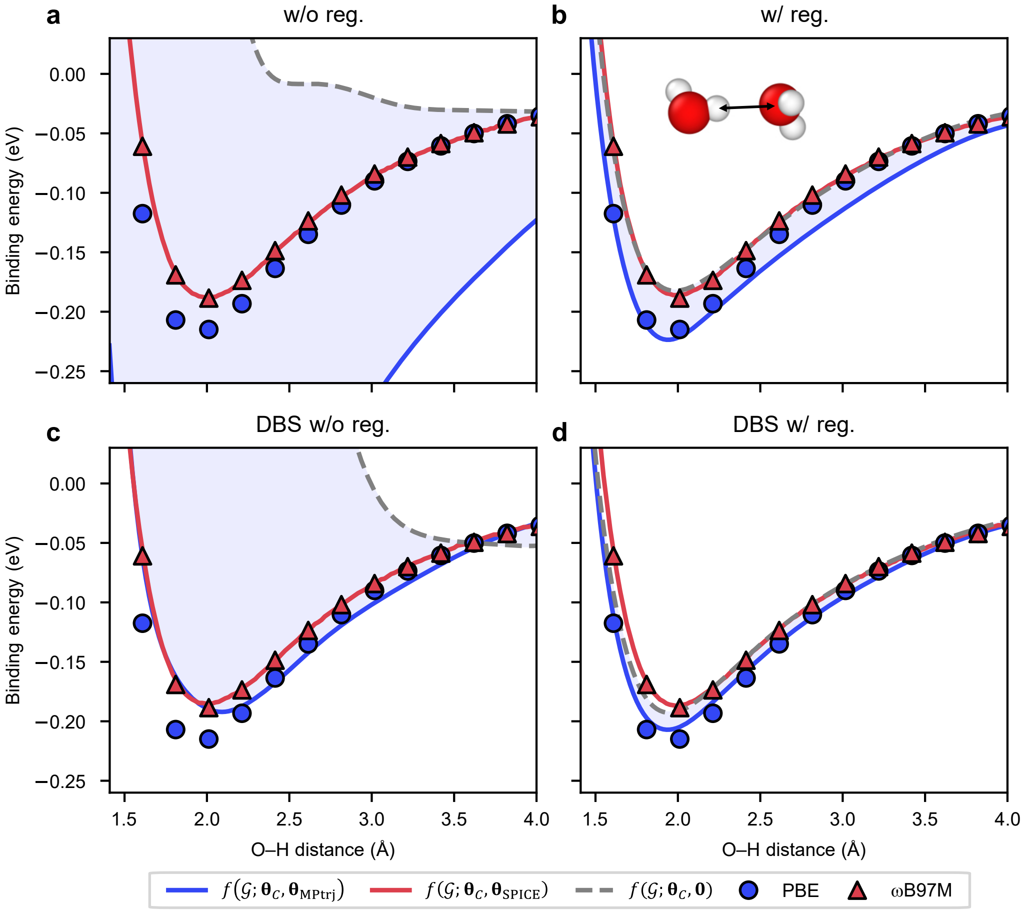

Supplementary Figure 2: **Potential energy surfaces predicted by multi-task SevenNet models trained using different strategies.** Blue and red solid lines show PES inferred with task-specific parameter trained on MPtrj ($\boldsymbol{\theta}_T = \boldsymbol{\theta}_{\mathrm{MPtrj}}$) and SPICE ($\boldsymbol{\theta}_T = \boldsymbol{\theta}_{\mathrm{SPICE}}$), respectively. Gray dashed lines show common PES obtained by explicitly setting task-specific parameters to zeros, while blue shaded region shows task-specific contribution of MPtrj channel. Circle and triangle markers indicate binding energy obtained from reference DFT calculation, employing PBE and ωB97M as XC functional, respectively. The first row (**a**, **b**) presents results for models trained on the MPtrj and SPICE databases without DBS, whereas the second row (**c**, **d**) shows the corresponding PES obtained with DBS. The left panels (**a**, **c**) and right panels (**b**, **d**) display results from models trained without and with regularization, respectively.

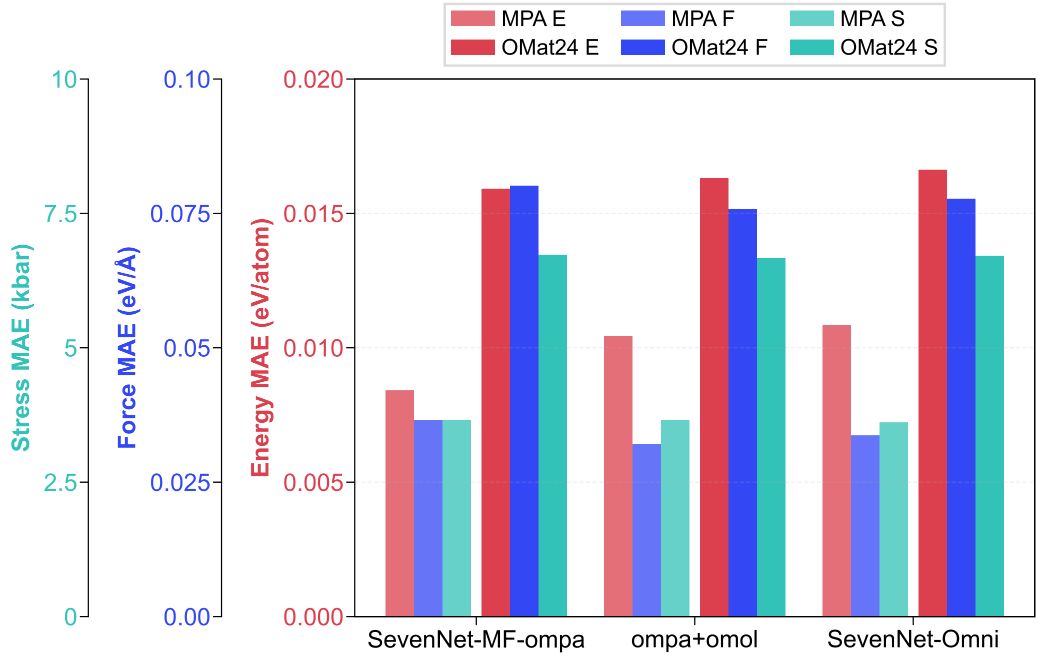

Supplementary Figure 3: **Prediction accuracies on crystal databases during curriculum learning.** Three models are compared: 7net-ompa, ompa+omol, and 7net-Omni. The ompa+omol denotes an intermediate model which is initialized from 7net-ompa and trained on MPtrj, sAlex, OMat24, and OMol25, along with DBS structures derived from OMol25 (see the Training section in the manuscript). MAEs of energies, forces, and stress components are evaluated on subsets of each training set. Specifically, the 'MPA' set is a subset of MPtrj+Alex database, and the 'OMat24' set is a subset of the OMat24 database. As curriculum learning progresses and the material domain of the training set is gradually expanded, the predictive accuracy on crystalline systems remains stable, showing no increase in training errors. The difference in the error balance between 7net-ompa and the other two models originates from the different force loss weights, where 7net-ompa used $\lambda_F = 0.1$ while others used $\lambda_F = 1$.

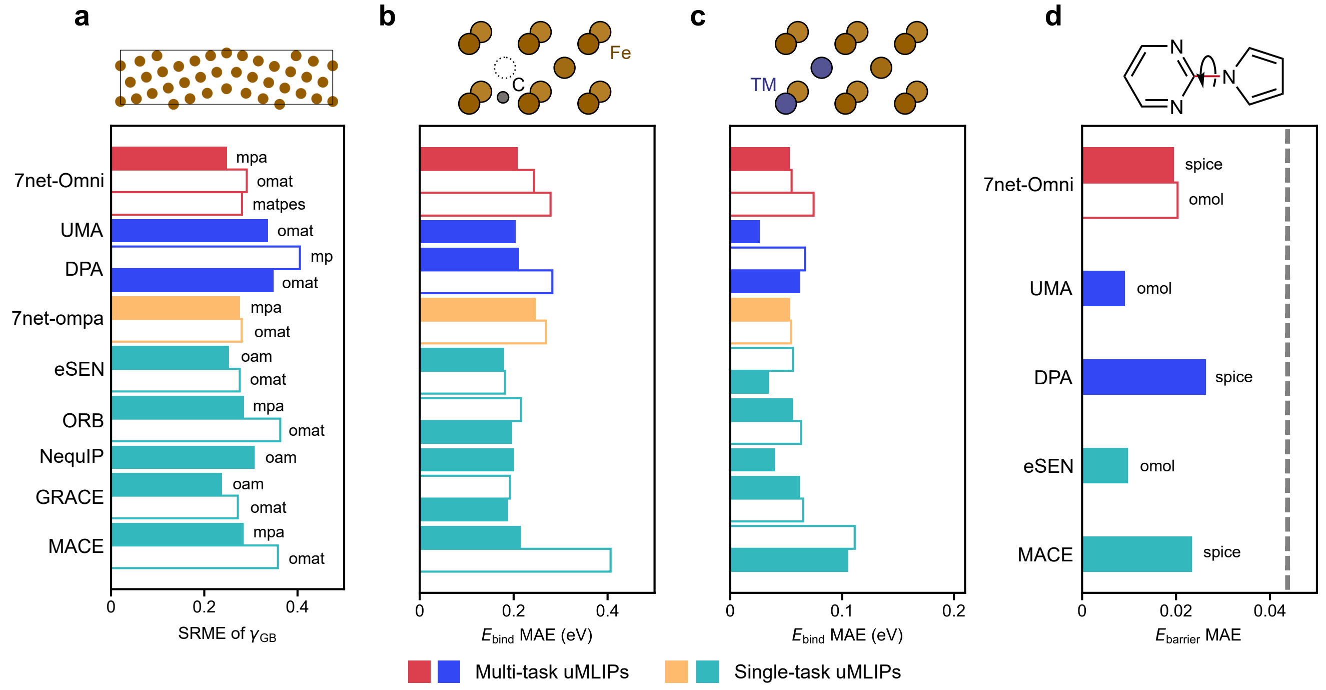

Supplementary Figure 4: **Performance of uMLIPs in single-domain tasks.** **a** SRME of grain boundary energies of elemental metals. **b** MAE of binding energies between carbon interstitials and vacancies in steels. **c** MAE of binding energies between transition metal solutes in steels. **d** MAE of torsional barriers. Gray dashed line denotes the error of 1 kcal/mol. Reference DFT data are calculated at the (**a**,**b**,**c**) PBE and (**d**) ωB97M-D3 levels of theory. Individual parity plots are presented in Supplementary Fig. 5 to 8.

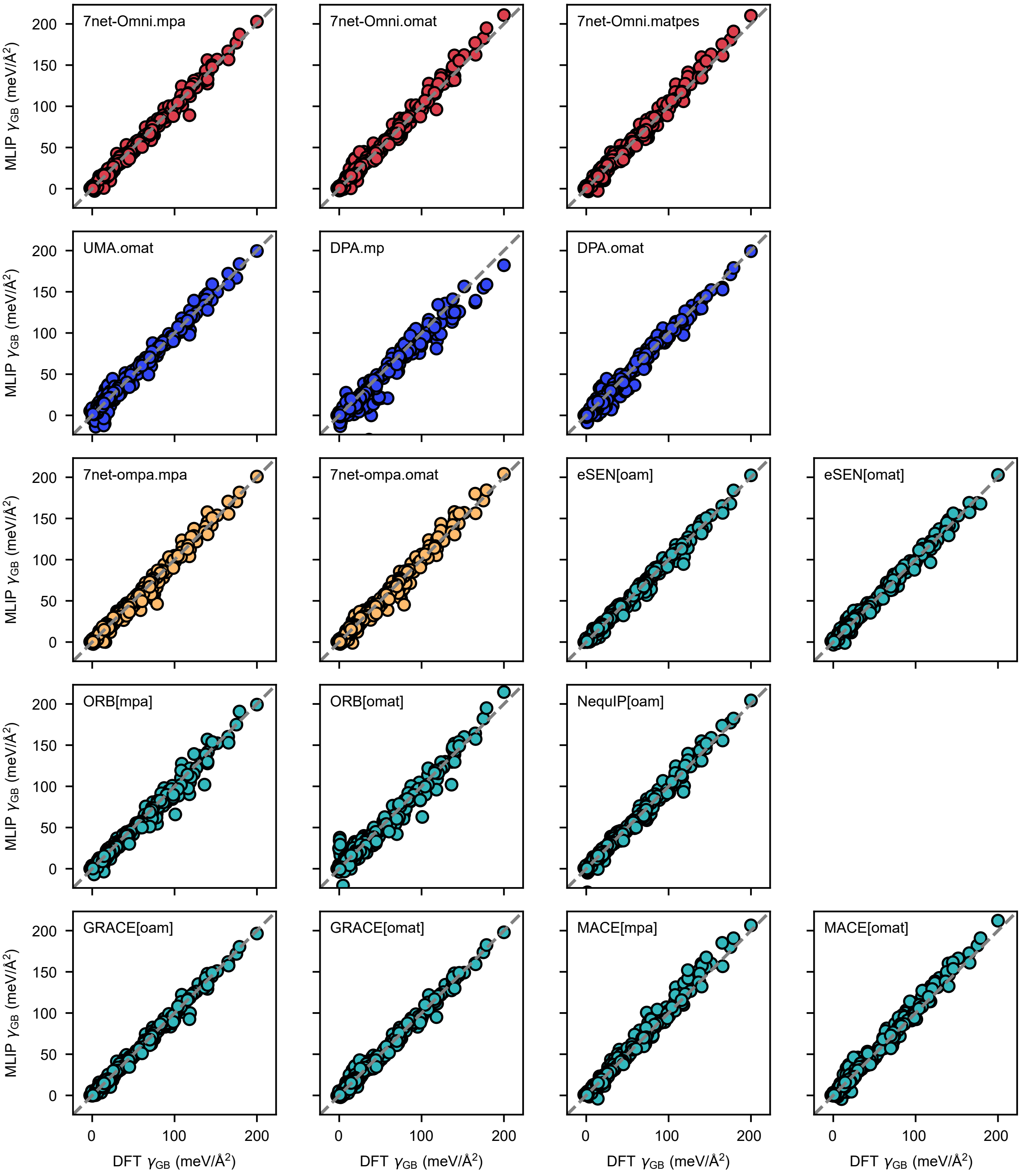

Supplementary Figure 5: **Parity plots of grain boundary energies of elemental metals.** Reference values are calculated using PBE functional.

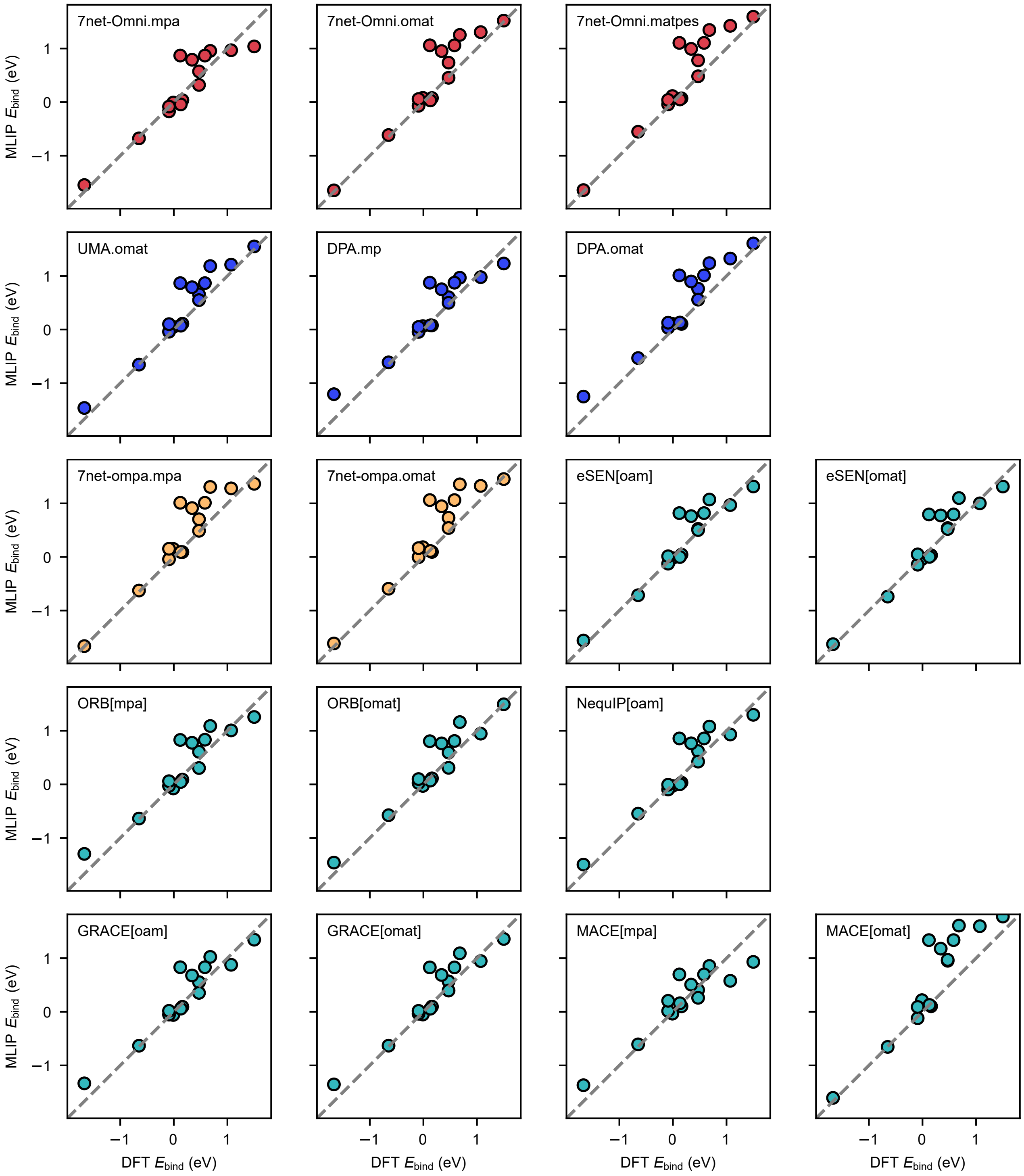

Supplementary Figure 6: **Parity plots of binding energies between carbon interstitials and vacancies in steels.** Reference values are calculated using PBE functional.

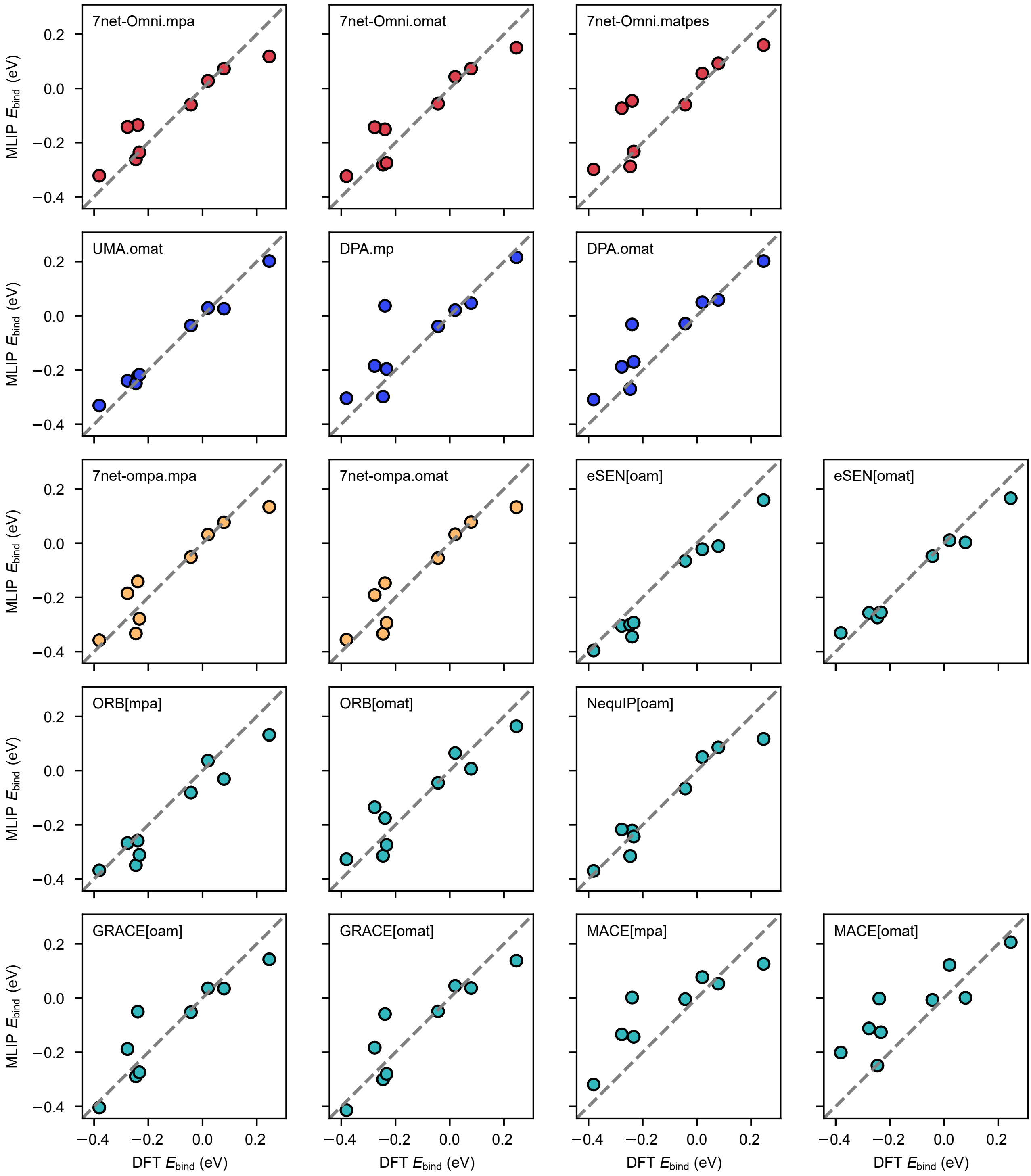

Supplementary Figure 7: **Parity plots of binding energies between transition metal solutes in steels.** Reference values are calculated using PBE functional.

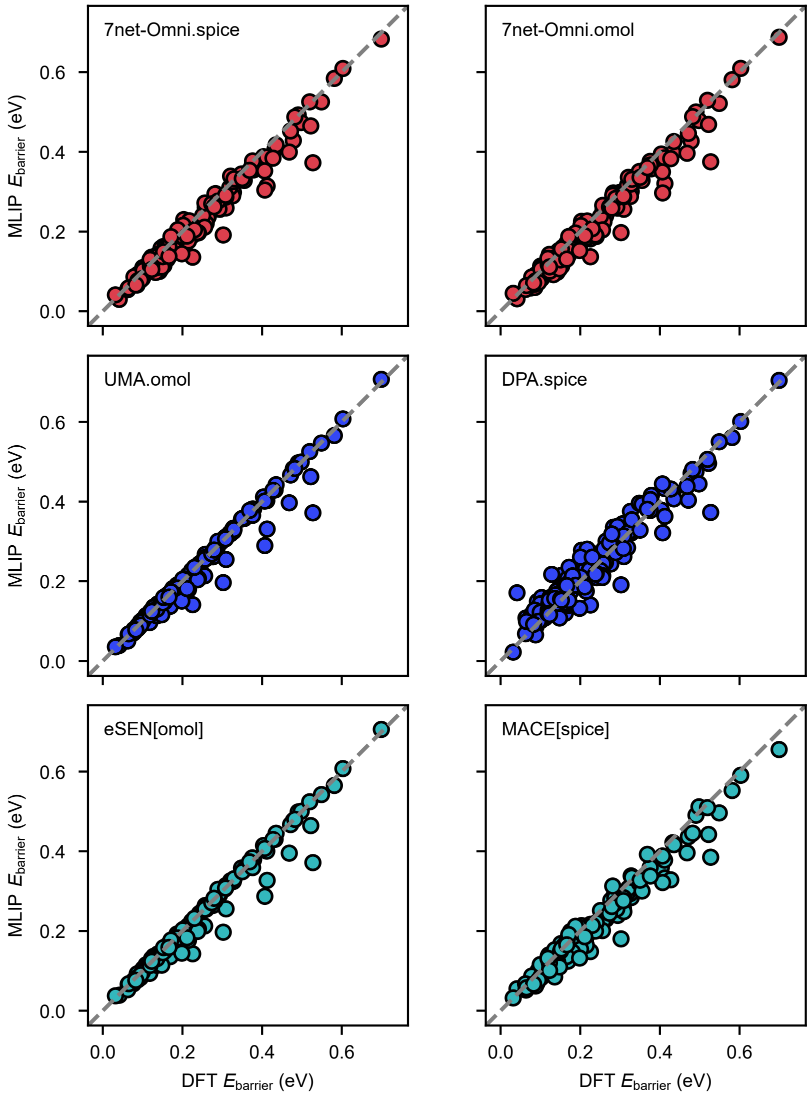

Supplementary Figure 8: **Parity plots of torsion barrier energies in molecules.** Reference values are calculated using ωB97M-D3 level of theory.

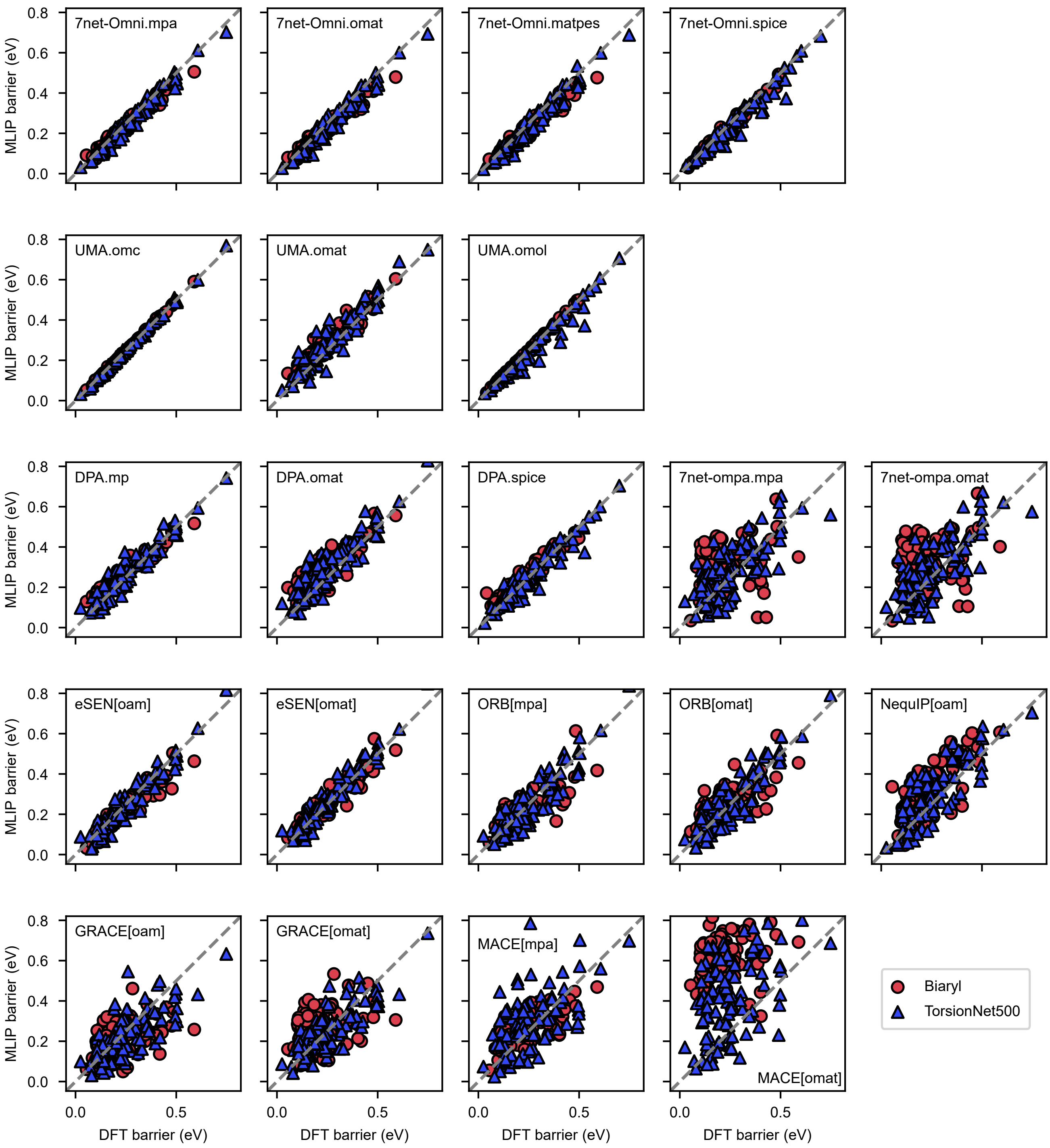

Supplementary Figure 9: **Parity plots of torsion barrier energies in molecules.** Reference values are calculated at the PBE-D3 level of theory, except for MLIPs employing hybrid-fidelity channels (e.g., 7net-Omni.spice), for which the ωB97M-D3 reference is used. Markers indicate the source of benchmark set at each data point (Biaryl or TorsionNet500).

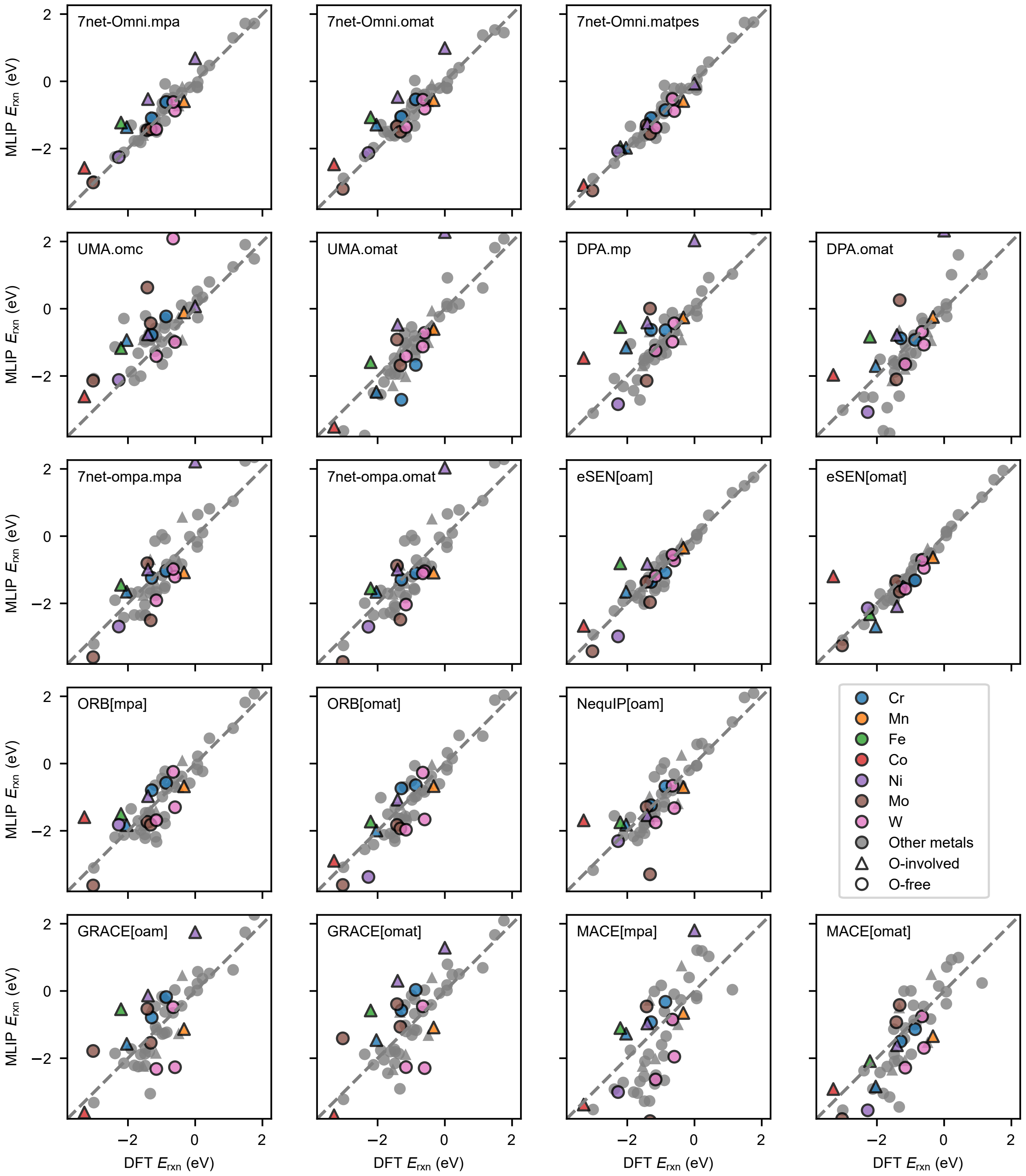

Supplementary Figure 10: **Parity plots of reaction energies on organometallic reaction benchmark.** Reference values are calculated at the PBE-D3 level of theory. Non-gray colors indicate reactions involving center metals for which Hubbard U correction is applied in the MPtrj/sAlex database, while other metals are shown in gray. Reactions where an oxygen atom is newly introduced near the center transition metal atom in the products are labeled as 'O-involved' (e.g. CO bonding to a core metal atom), whereas reactions in which the number of oxygen atoms near the metal center remains unchanged are labeled as 'O-free', The predictive accuracy exhibits metal-dependent variations, with O-involved reactions at Ni, Cr, Fe, and Co centers showing relatively large deviations in reaction energies in 7net-Omni.mpa. In constrast, matpes channel presents more uniform accuracy across the metal centers.

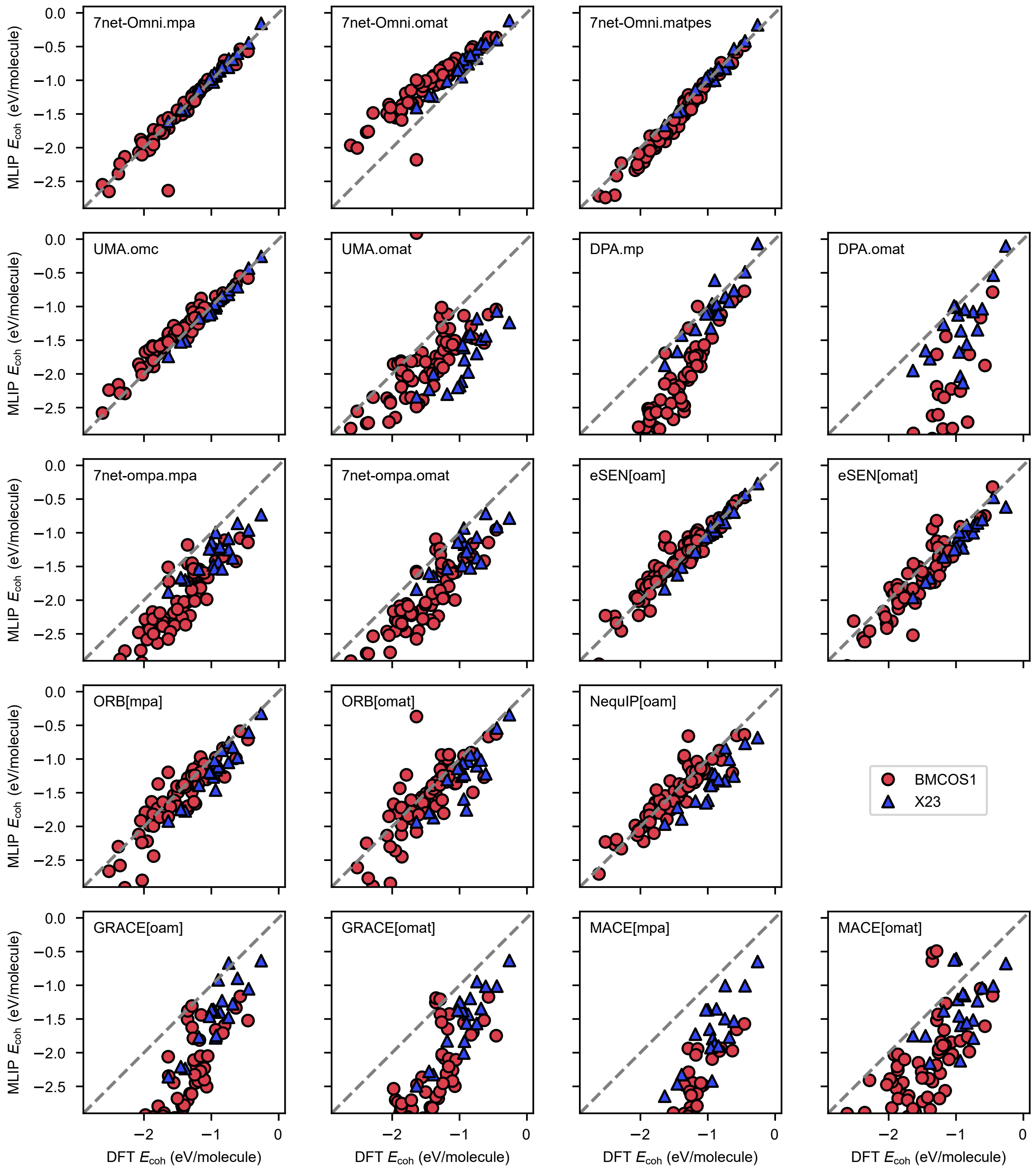

Supplementary Figure 11: **Parity plots of cohesive energies of molecular crystals.** Reference values are calculated at the PBE-D3 level of theory. Markers indicate the source of benchmark set at each data point (BMCOS1 or X23).

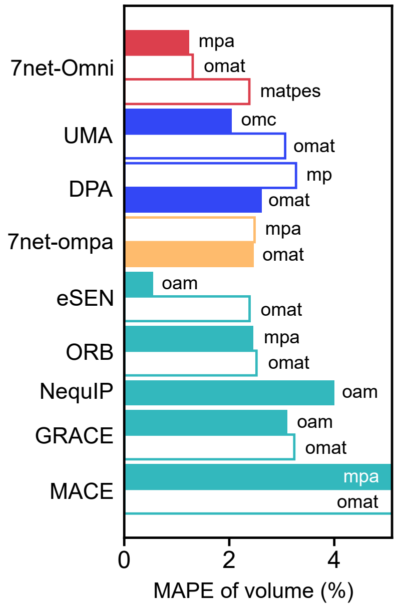

Supplementary Figure 12: **Volume prediction results for molecular crystals.** Mean absolute percentage errors (MAPEs) of volume predictions by uMLIPs for 86 molecular crystals in the X23 and BMCOS1 benchmark sets. Parity plots for each uMLIP are presented in Supplementary Fig. 13.

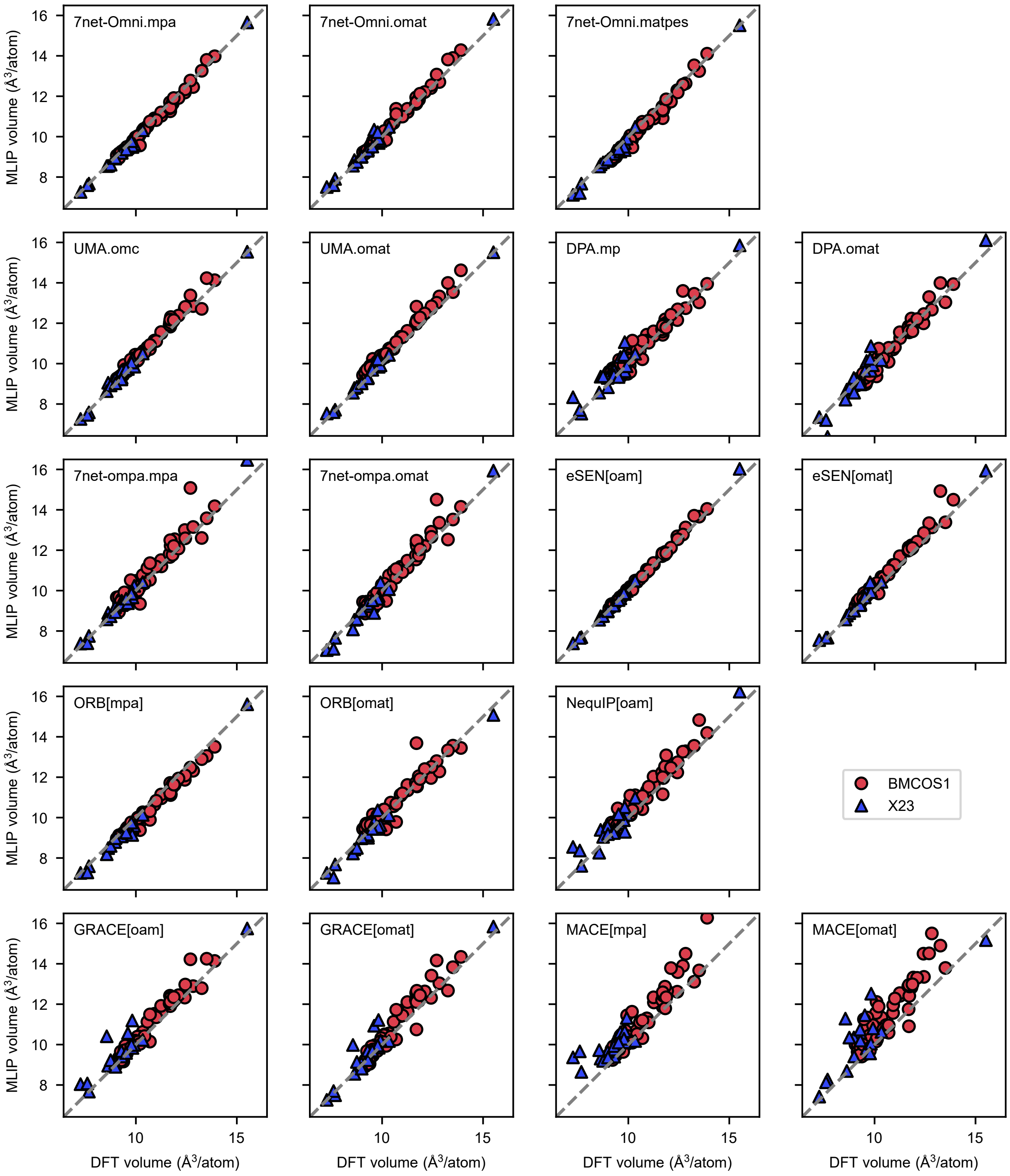

Supplementary Figure 13: **Parity plots of equilibrium volumes of molecular crystals.** Reference values are calculated at the PBE-D3 level of theory. Markers indicate the source of benchmark set at each data point (BMCOS1 or X23).

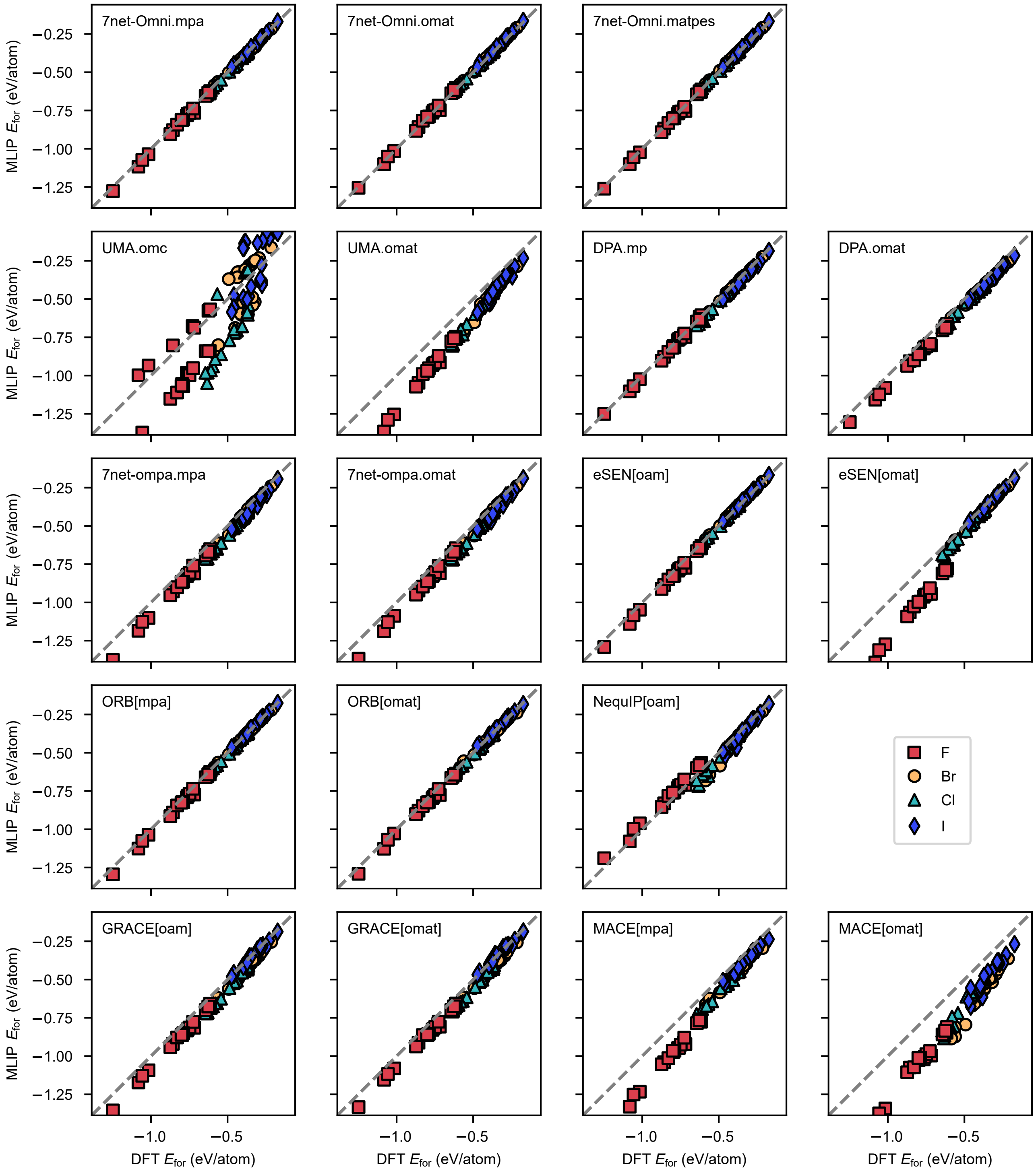

Supplementary Figure 14: **Parity plots of relative energies of hybrid perovskites.** Reference values are calculated at the PBE-D3 level of theory. Markers indicate the halogen element contained in the perovskite.

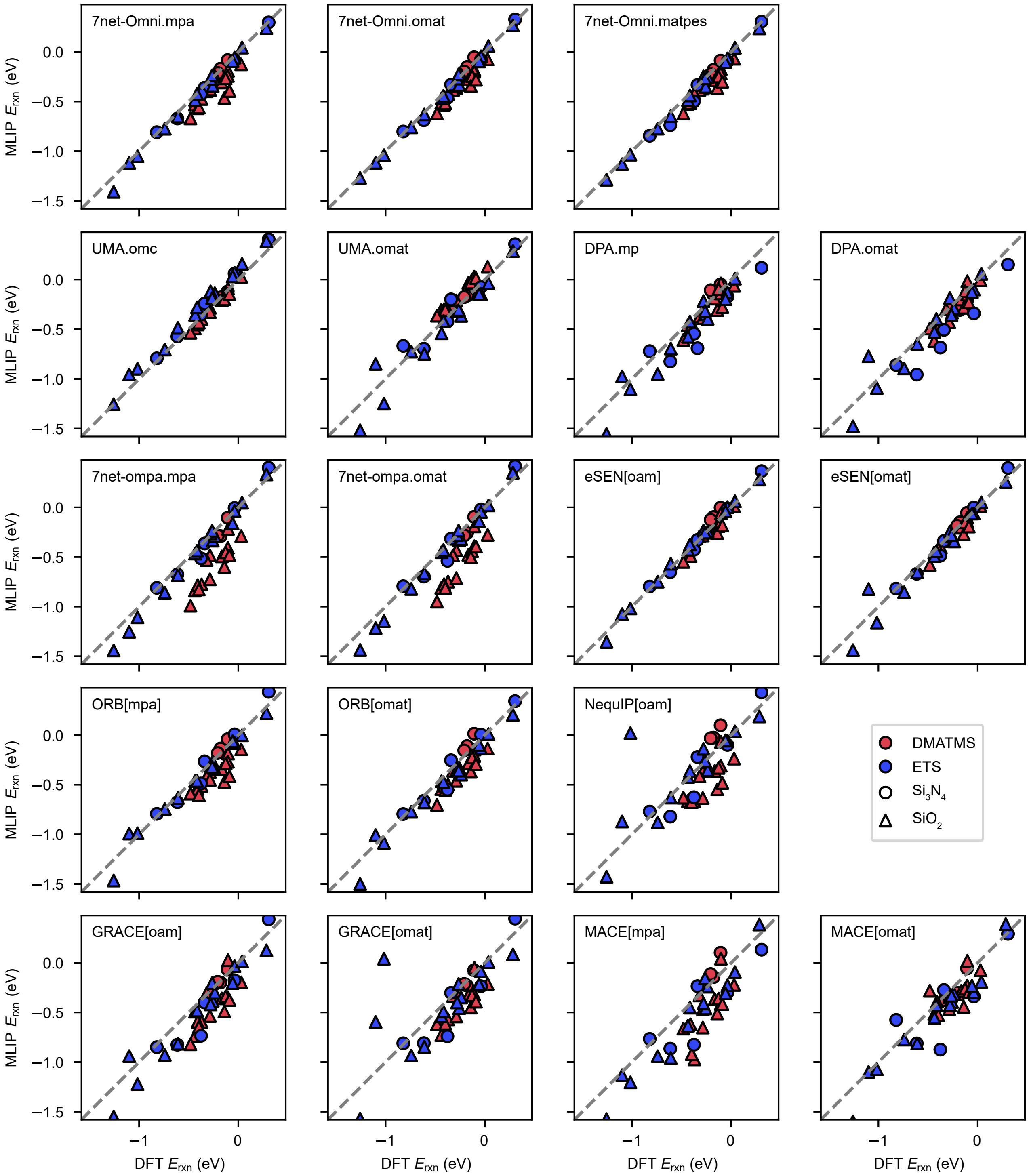

Supplementary Figure 15: **Parity plots of reaction energies in area-selective deposition benchmark.** Reference values are calculated at the PBE-D3 level of theory. Color of each marker indicates the type of inhibitor molecule, while circle and triangle marker corresponds to Si_3N_4 and SiO_2 surface, respectively.

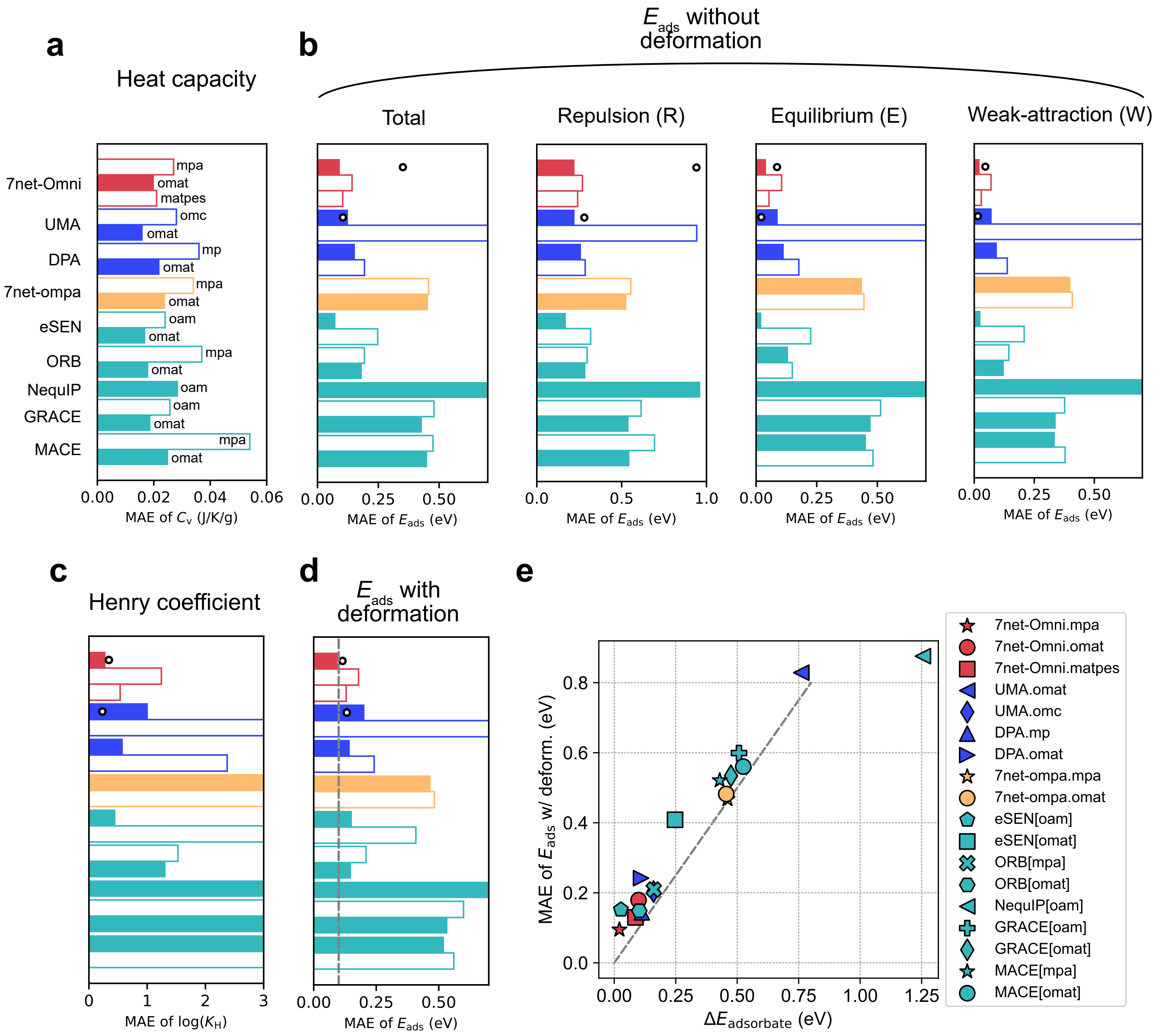

Supplementary Figure 16: **Benchmark tasks results for metal-organic frameworks. a** MAE of heat capacity. **b** MAE of adsorption energy without deformation. Since the GoldDAC dataset can be separated by its adsorption type, MAEs according to three different (repulsion, equilibrium, and weak-attractive) cases were displayed. **c** MAE of CO_2 henry coefficient. Logarithm value of henry coefficient were used for convenience. **d** MAE of adsorption energy with deformation. Gray dashed line denotes 0.1 eV. **e** The relationship between error in energy of adsorbate and MAE of adsorption energy with deformation. Gray dashed line denotes $y = x$. Bulltin points in Fig. **b-d** respectively denote 7net-Omni.odac23 and UMA.odac. Individual parity plots are presented in Supplementary Fig. 18 to 22.

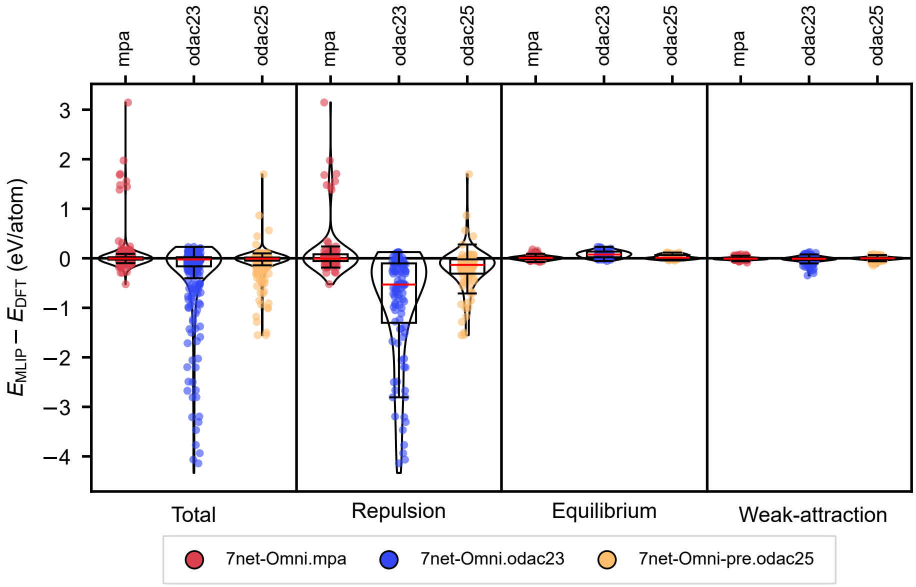

Supplementary Figure 17: **Adsorption energy error without deformation in metal-organic frameworks.** Error distribution is illustrated using violin and box plots. The box and red horizontal line in box plot shows quartiles and median of the error distribution, respectively while whiskers represent 1.5 times the inter-quartile range. Red, blue and yellow markers indicate adsorption energy difference between DFT and uMLIP, employing `mpa` channel of 7net-Omni, `odac23` channel of 7net-Omni, and `odac25` channel of 7net-Omni-pre, respectively. 7net-Omni-pre is a preliminary model that are trained on subsampled ODAC25 database instead of ODAC23 database utilized in original 7net-Omni, but without updating DBS. Individual data points are randomly jittered along the horizontal axis for visual clarity.

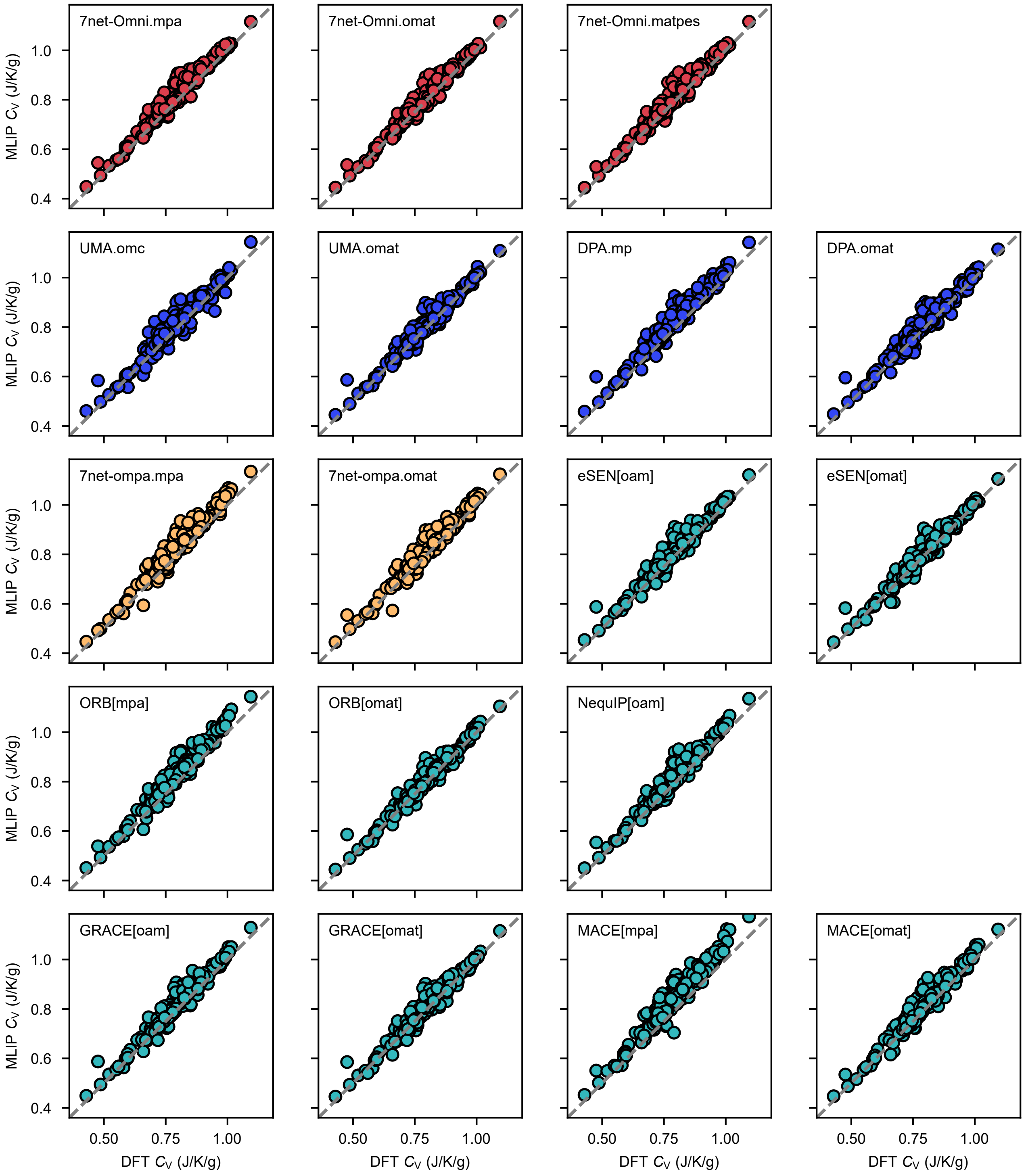

Supplementary Figure 18: **Parity plots of heat capacities of MOFs.** Reference values are calculated at the PBE-D3 level of theory.

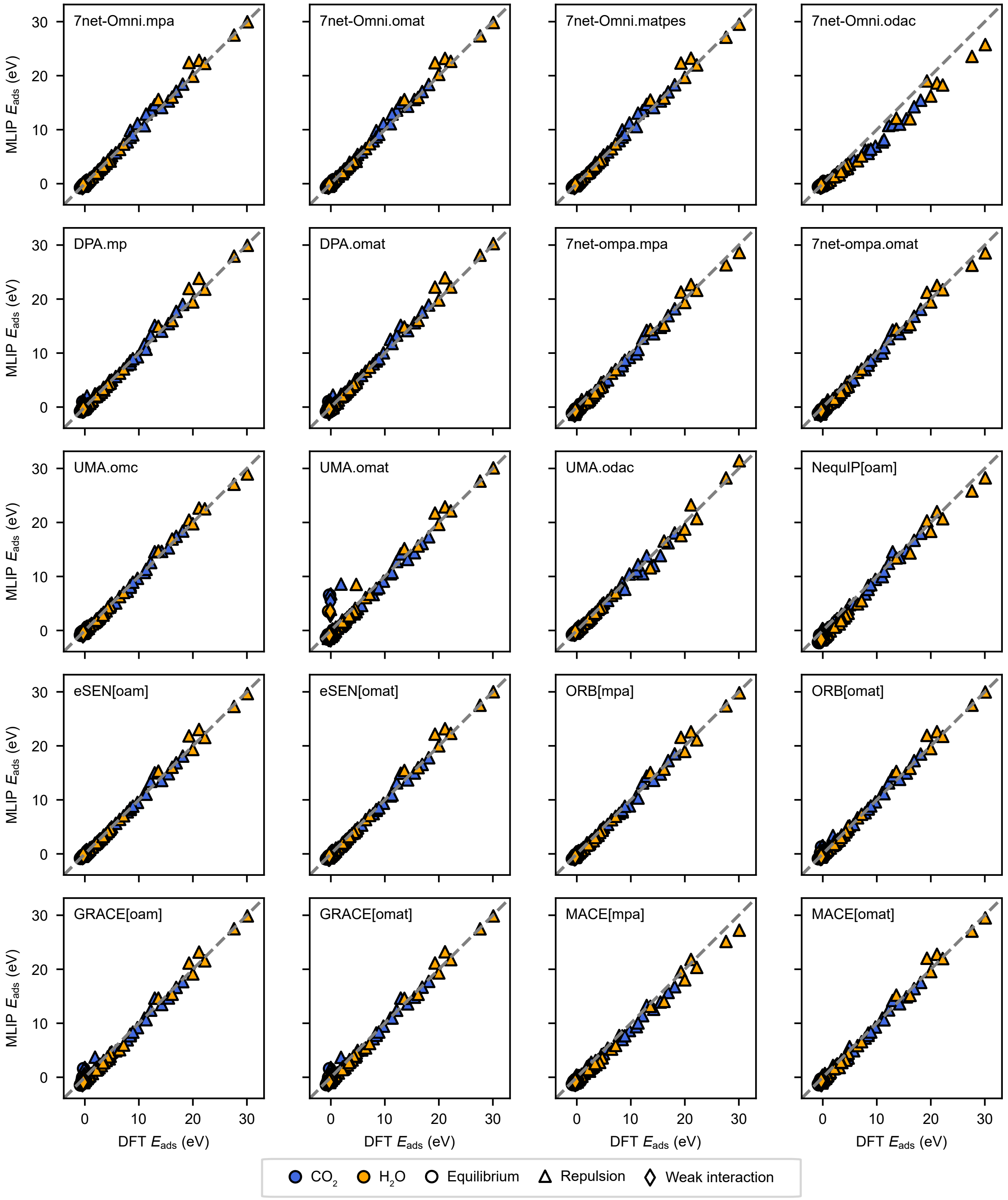

Supplementary Figure 19: **Parity plots of adsorption energy of molecules in MOFs without relaxation.** Reference values are calculated at the PBE-D3 level of theory. Color of each marker indicates the type of molecule (CO_2 and H_2O), while circle, triangle and diamond marker corresponds to energy region of equilibrium, repulsion, and weak interaction, respectively.

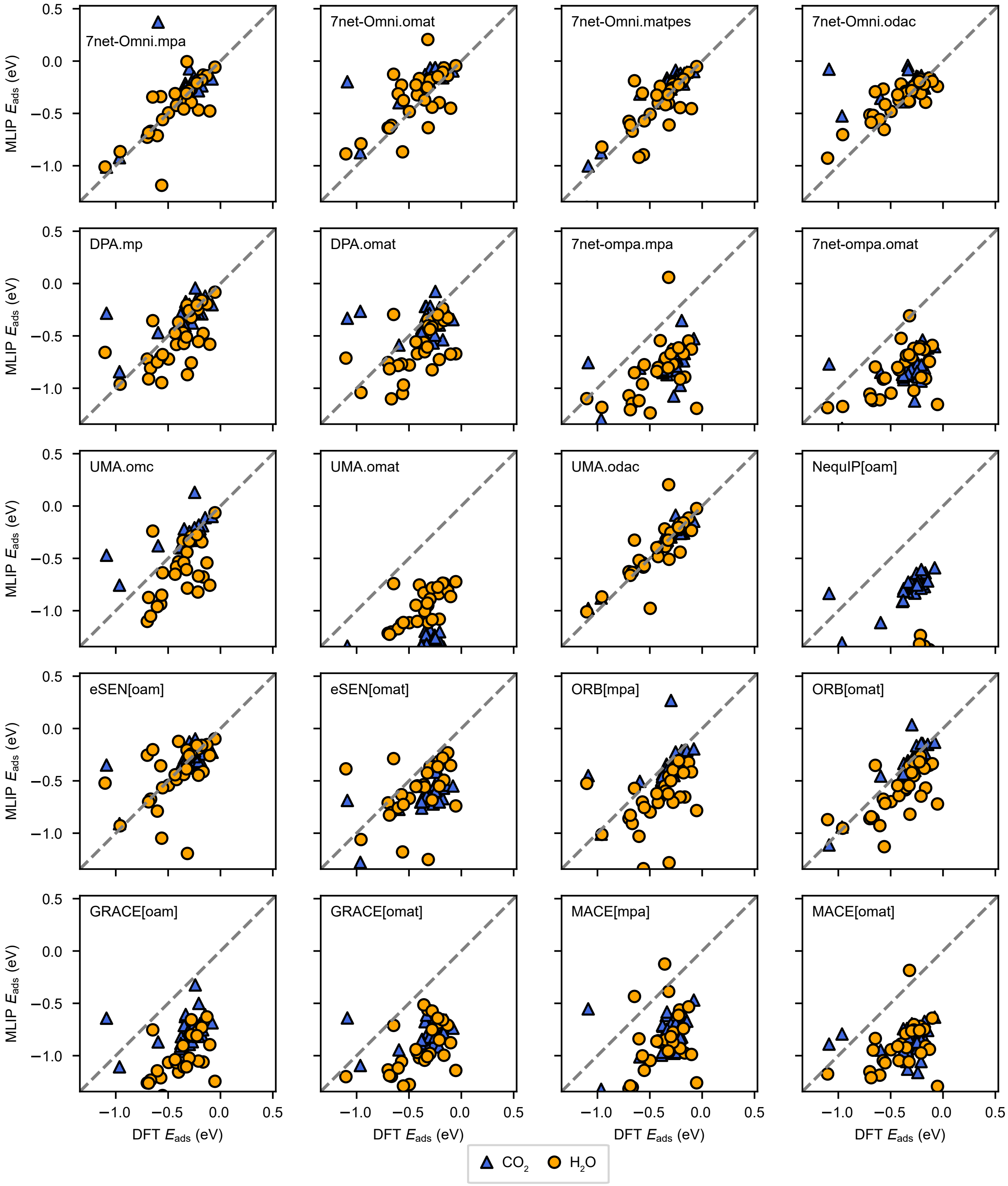

Supplementary Figure 20: **Parity plots of adsorption energy of molecules in MOFs with relaxation.** Reference values are calculated at the PBE-D3 level of theory. Color of each marker indicates the type of molecule (CO_2 and H_2O) adsorbed to the MOF.

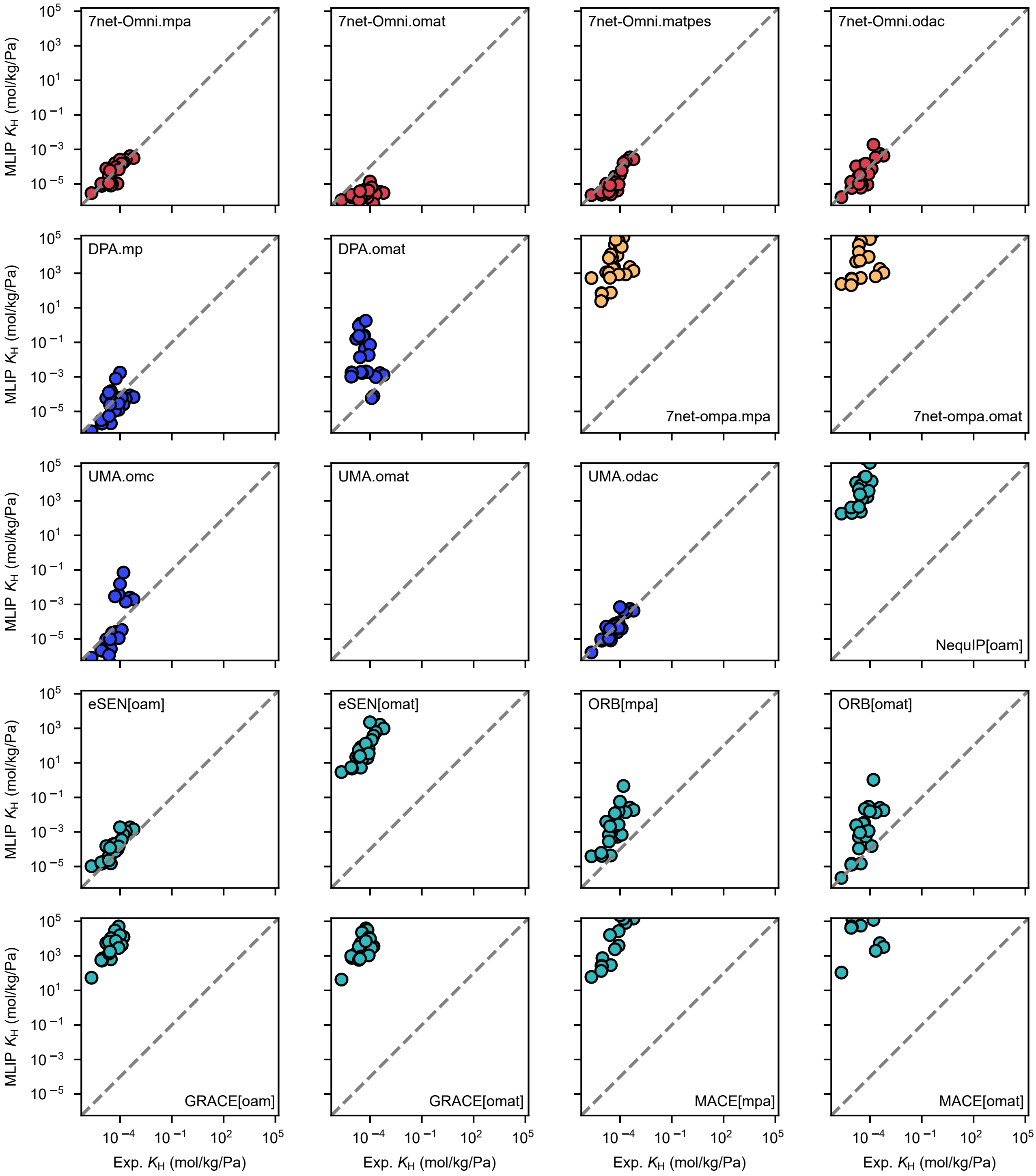

Supplementary Figure 21: Parity plots of Henry coefficients of MOFs. Reference values are obtained by experiment.

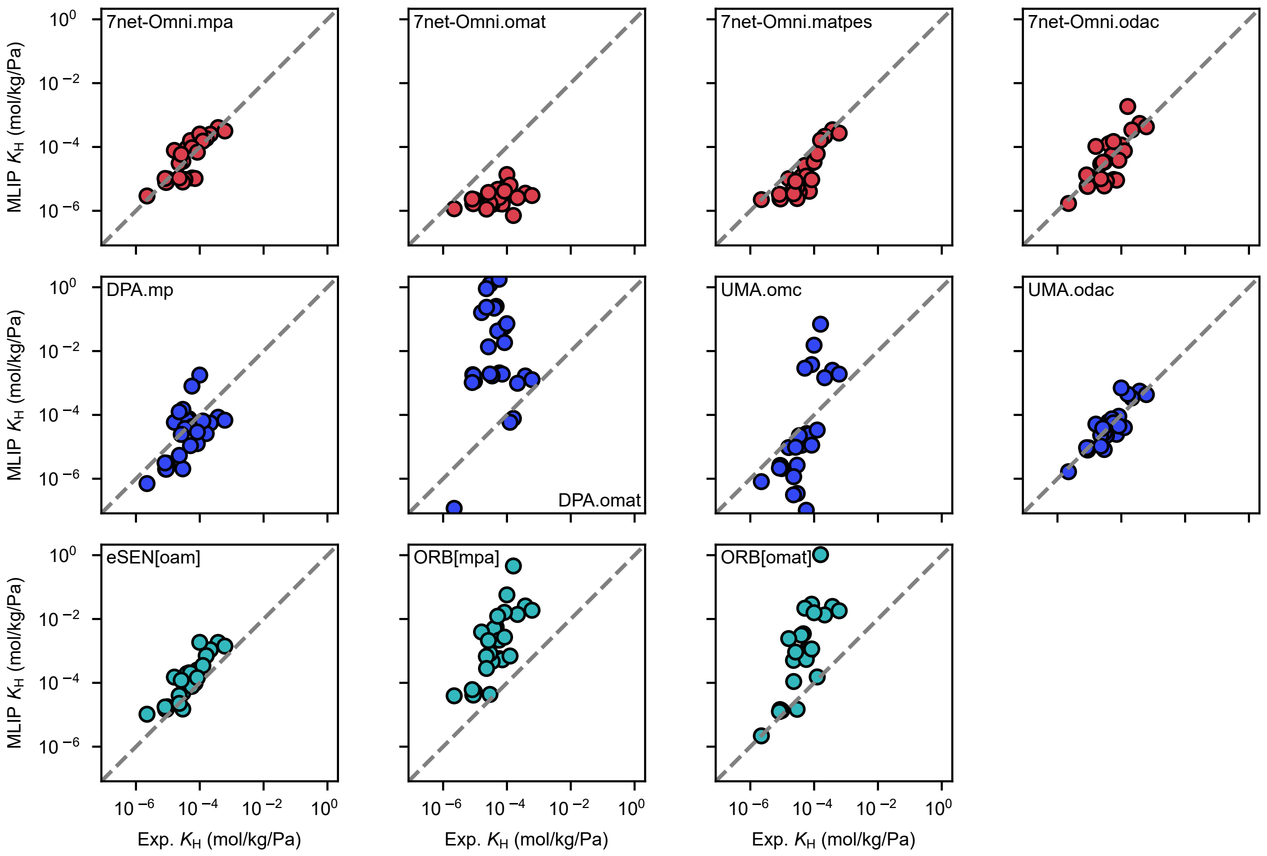

Supplementary Figure 22: **Parity plots of Henry coefficients of MOFs, only showing MLIPs that give reasonable range of results.** Reference values are obtained by experiment.

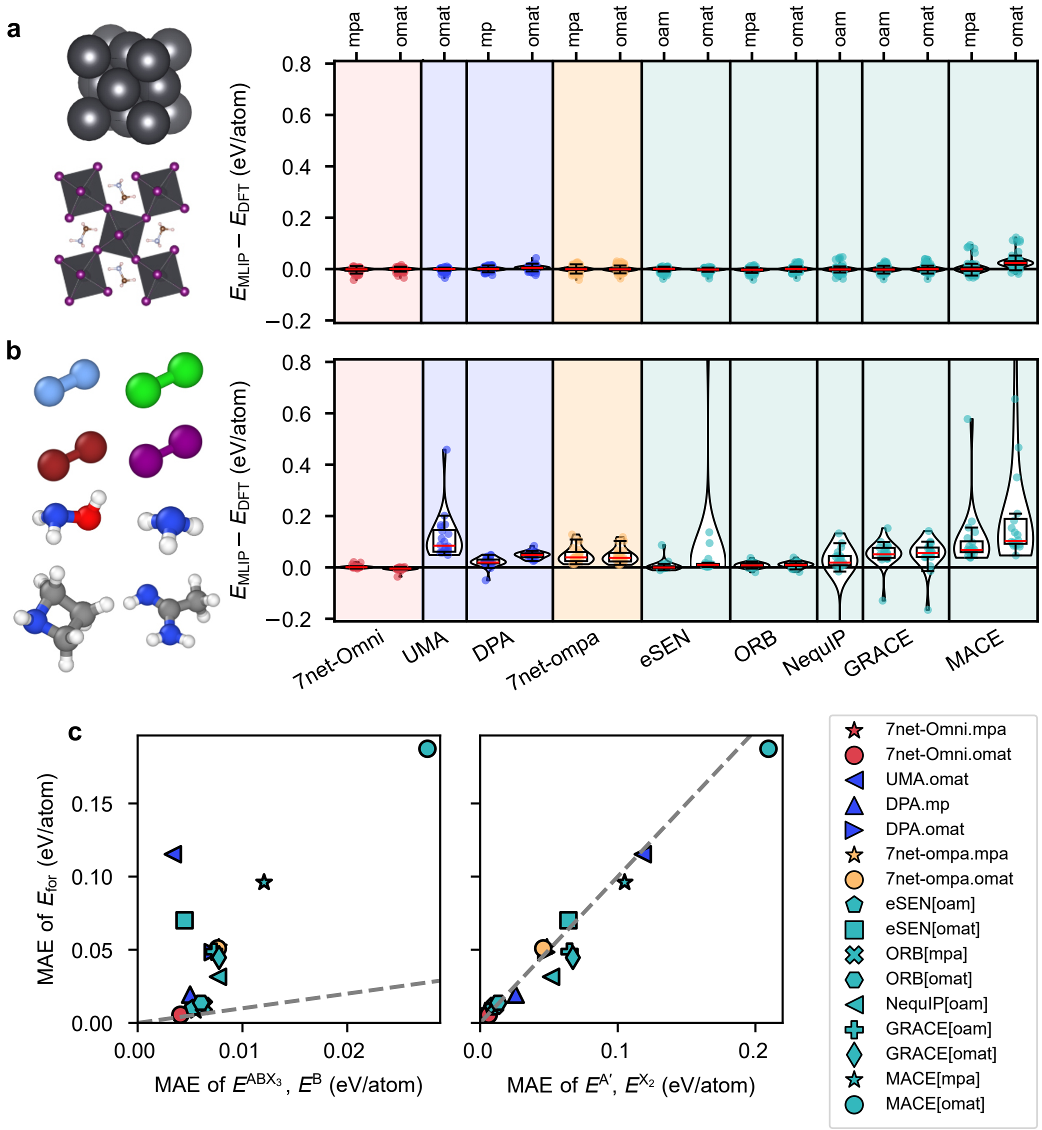

Supplementary Figure 23: **Error analysis of the atomic structures related to hybrid organic-inorganic perovskite systems. a,b** Distribution of total energy difference between uMLIPs and respective DFT calculations, illustrated with violin and box plots. DFT total energy is obtained by the consistent calculation settings and pseudopotentials utilized in each corresponding training set. The box and red horizontal line in box plot shows quartiles and median of the error distribution, respectively while whiskers represent 1.5 times the inter-quartile range. Individual data points are randomly jittered along the horizontal axis for visual clarity. **a** and **b** show error distribution of the bulk systems ($E^{\mathrm{ABX_3}}$ and E^{B}) and molecular systems ($E^{\mathrm{A'}}$ and $E^{\mathrm{X_2}}$), respectively. **c** Correlation between MAE of total energy and MAE of E_{for}. Gray dashed lines indicate $y = x$.

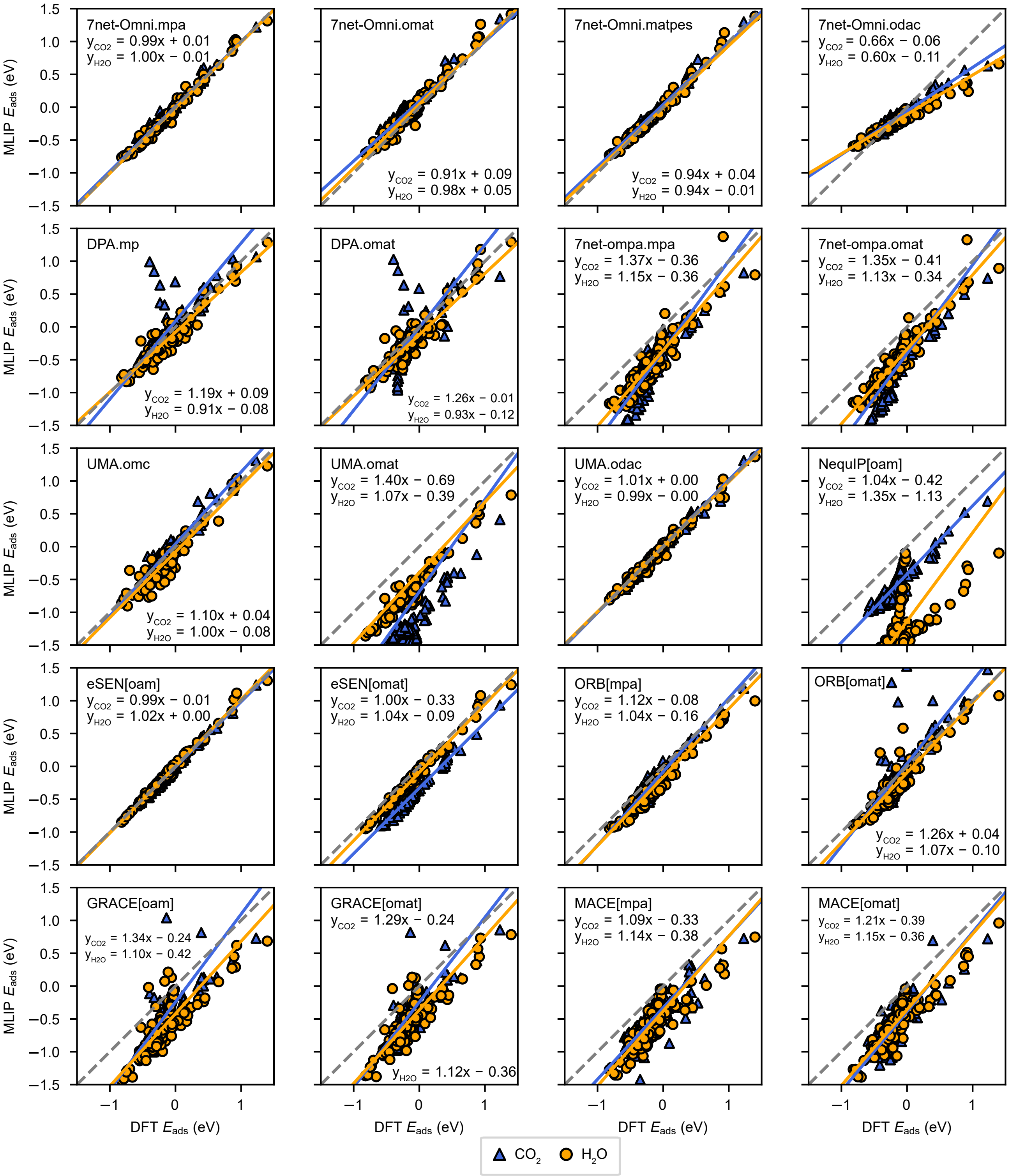

Supplementary Figure 24: **Parity plots of adsorption energy of molecules in MOFs without relaxation, enlarged to the energy region under 1.5 eV.** Reference values are calculated at the PBE-D3 level of theory. Blue and orange lines indicate linear regression line fitted to the data points of DFT adsorption energy less than 0.5 eV, corresponds to CO_2 and H_2O, respectively.

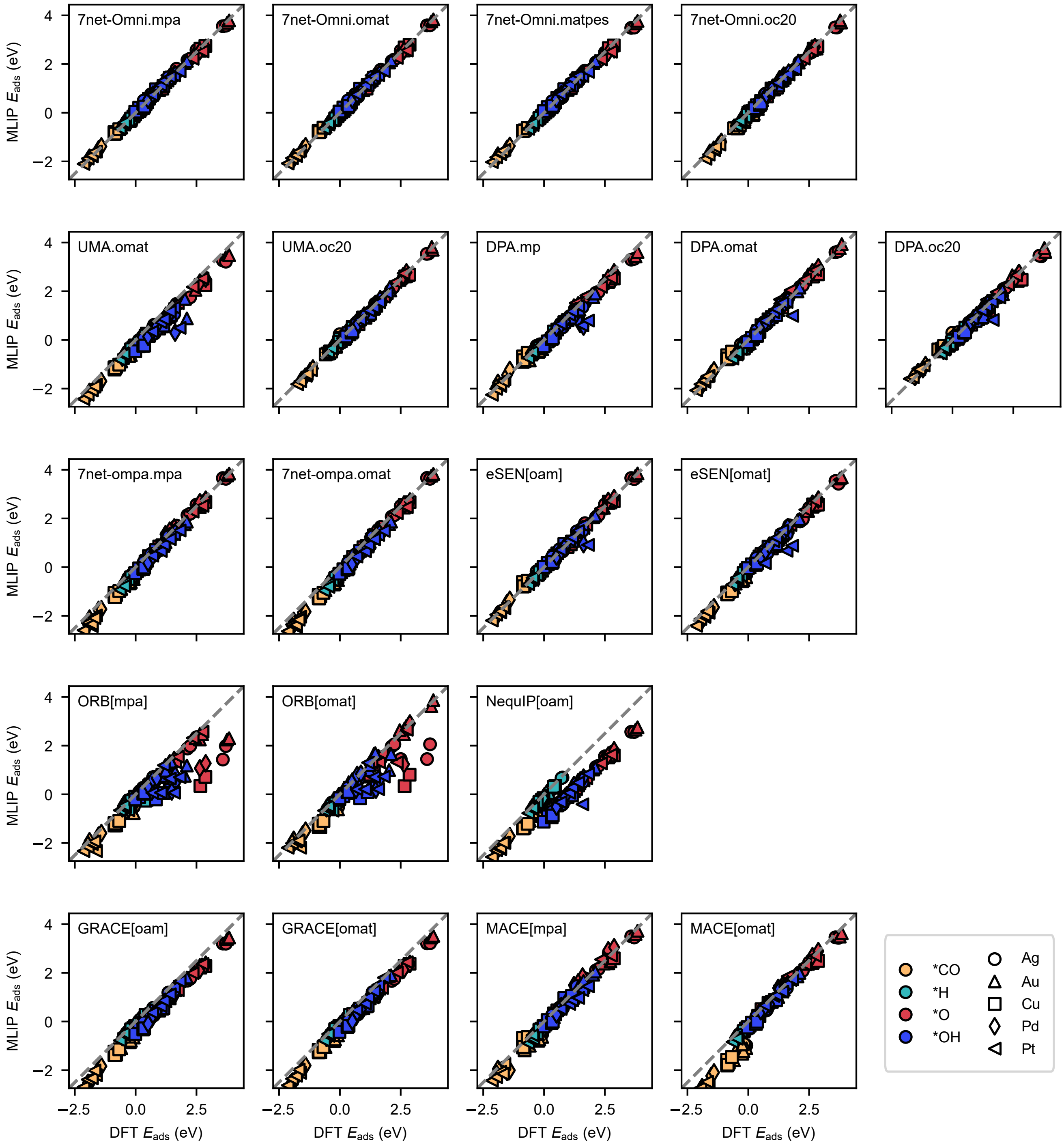

Supplementary Figure 25: **Parity plots of adsorption energies in noble metal surfaces.** Reference values are calculated with PBE functional except for MLIPs employing RPBE-fidelity channels (e.g., 7net-Omni.oc20), for which the RPBE reference is used. Color of each marker indicates the type of adsorbates, while shape of marker corresponds to the type of noble metal.

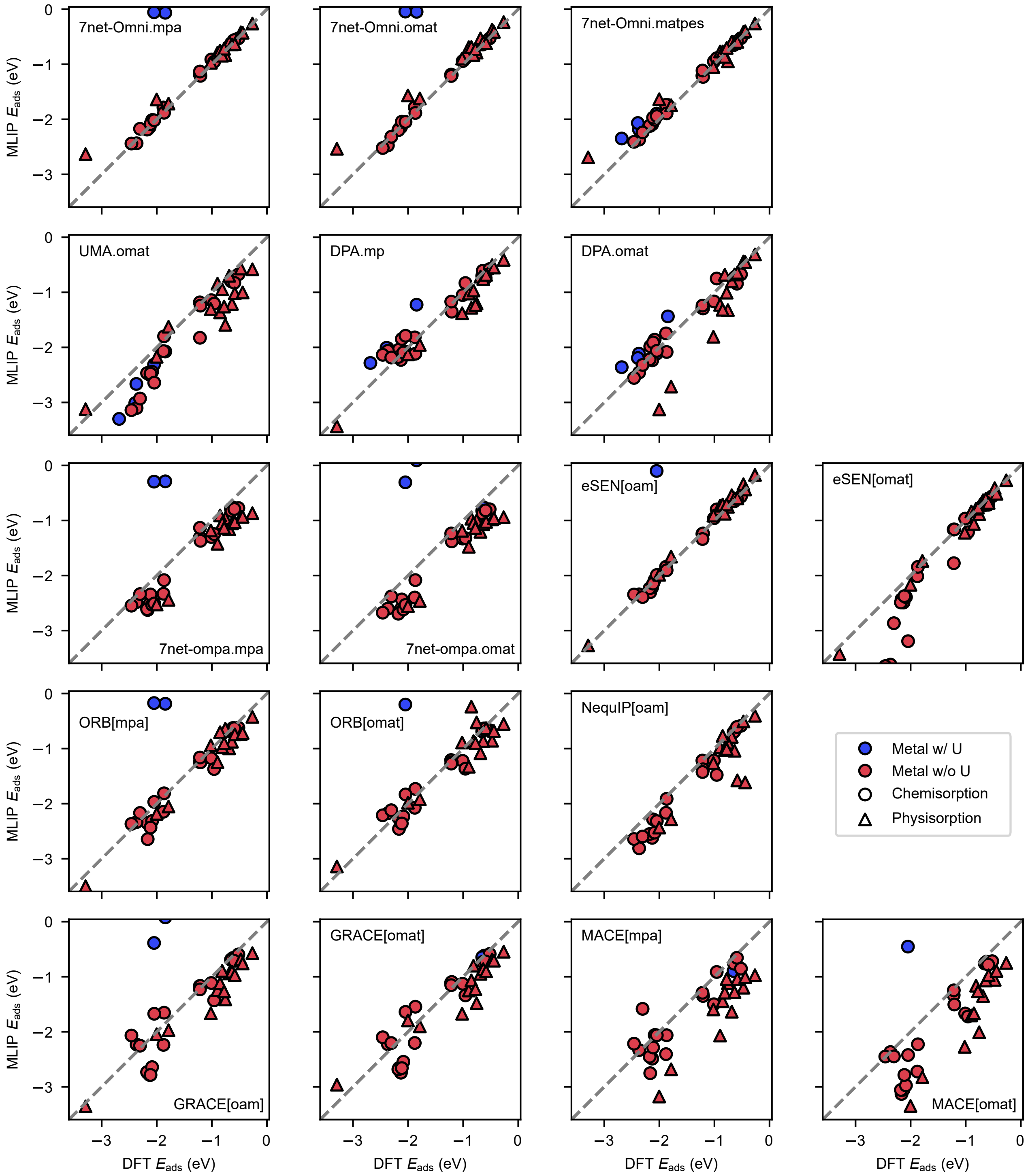

Supplementary Figure 26: **Parity plots of adsorption energies in ADS41 benchmark.** Reference values are calculated with PBE-D3 level of theory. Color of marker indicates whether corresponding metal element utilizes Hubbard U correction in MPtrj/sAlex database, while circle and triangle marker corresponds to chemisorption and physisorption, respectively.

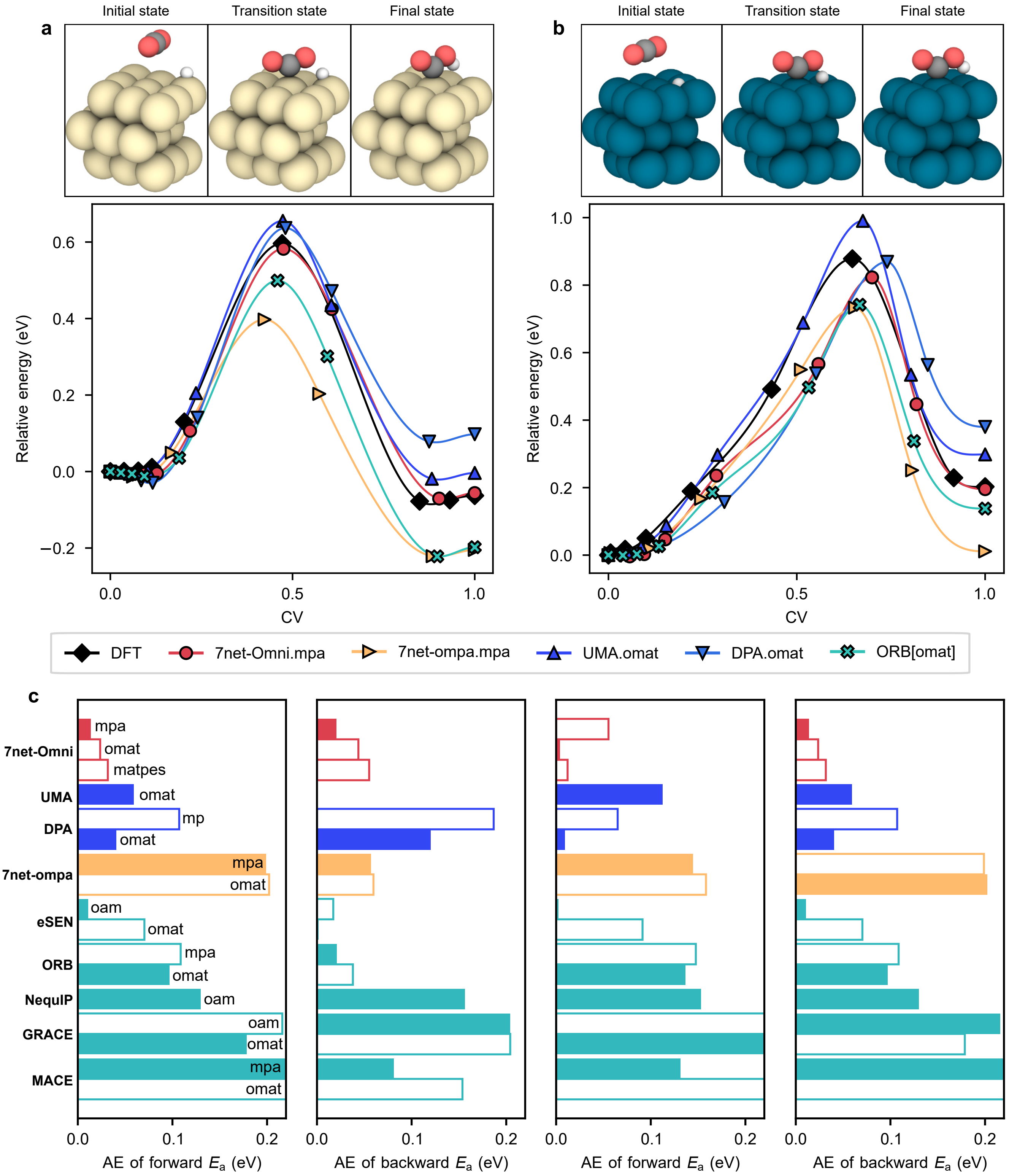

Supplementary Figure 27: **Benchmark results in CO_2RR on Pt and Pd surfaces. a** NEB calculation results in Pt (111) surface. PES are illustrated using collective variable (CV) based on distance between carbon atom in CO_2 and surface Pt atom, as well as hydrogen atom and surface Pt atom. **b** Similar PES for Pd (111) surface. **c** Absolute error (AE) in forward and backward activation barrier energy (E_a). Left two panels indicate results for Pt surface while right two panels show results for Pd surface.

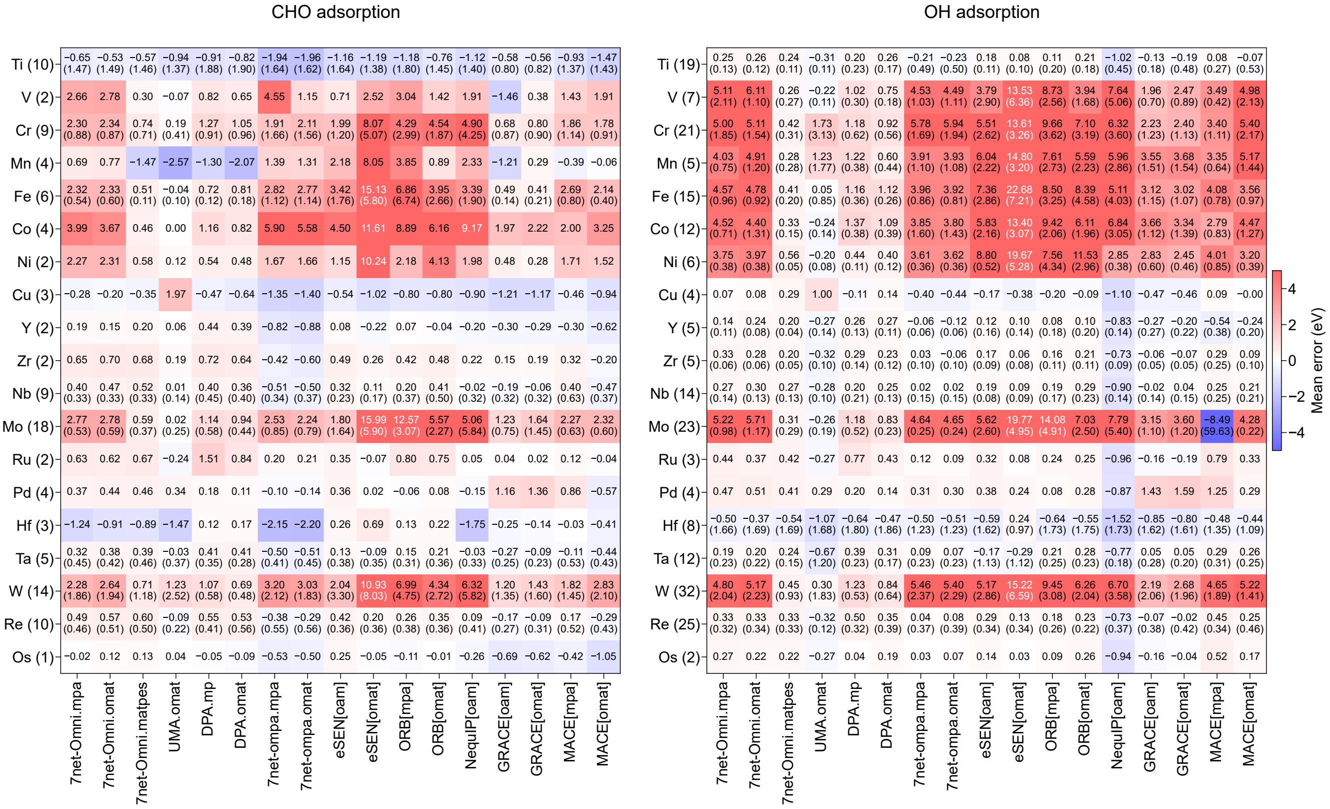

Supplementary Figure 28: **Adsorption on transition metal nitrides.** Heatmap of mean errors (eV) in adsorption energies for CHO (left) and OH (right) adsorption on transition metal nitrides, calculated using different uMLIPs. Each row corresponds to a transition metal element, where the total number of adsorption reactions for each metal is indicated in parentheses to the right of the element. Standard deviations are shown below the mean error values when the number of reactions exceeds four. The color and its intensity denotes the sign and magnitude of the mean error, respectively. Reference adsorption energies at the PBE level of theory and corresponding geometries are taken from ref. [3]. For each adsorbate–slab pair, the most stable configuration is selected. Most models show spurious overestimation of the adsorption energy for oxygen-containing adsorbates on nitrides of U-corrected transition metals, except for 7net-Omni.matpes, UMA, and DPA.

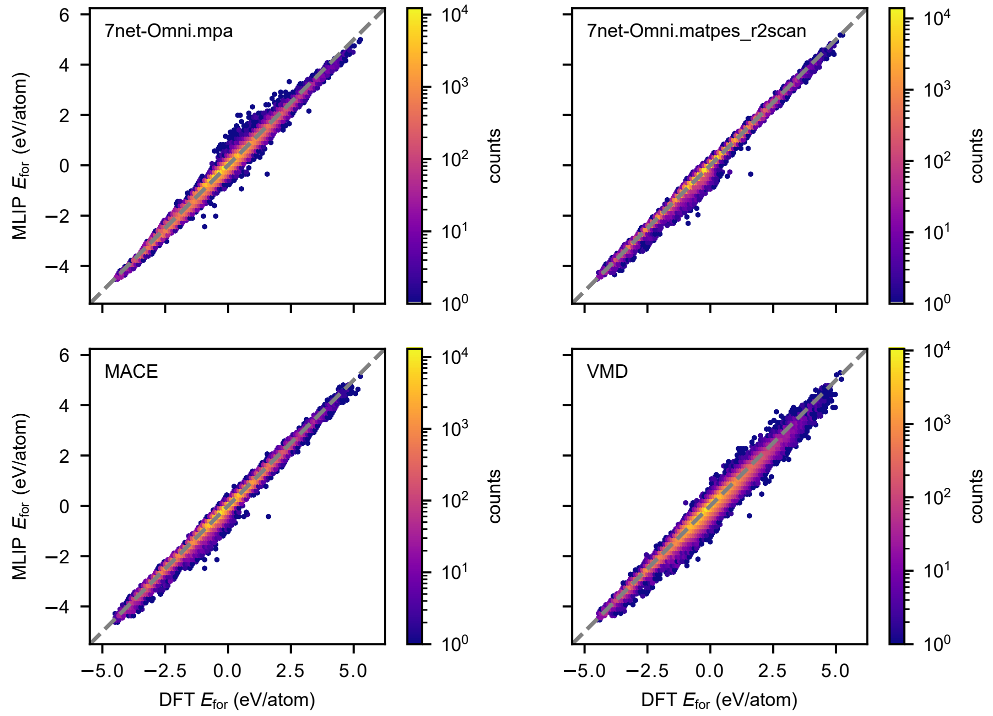

Supplementary Figure 29: **Parity plots of formation energies of inorganic crystals.** Reference values are calculated with meta-GGA SCAN functional for the structures relaxed with PBEsol functional. Color at each region indicates the count of data points. To calculate formation energy from 7net-Omni.mpa, we utilized energy correction suggested in ref. [4], which employs mixing scheme between GGA and GGA+U.

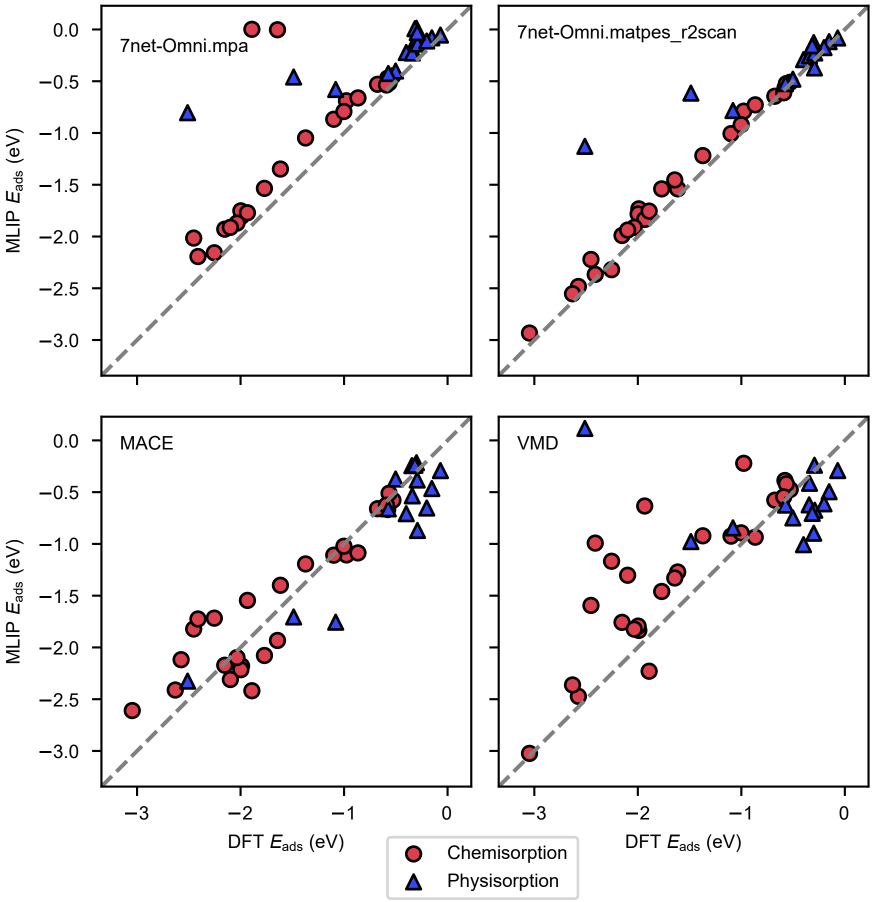

Supplementary Figure 30: **Parity plots of adsorption energies in ADS41 benchmark.** Reference values are calculated with meta-GGA r^{2}SCAN functional. Circle and triangle markers indicate chemisorption and physisorption, respectively.

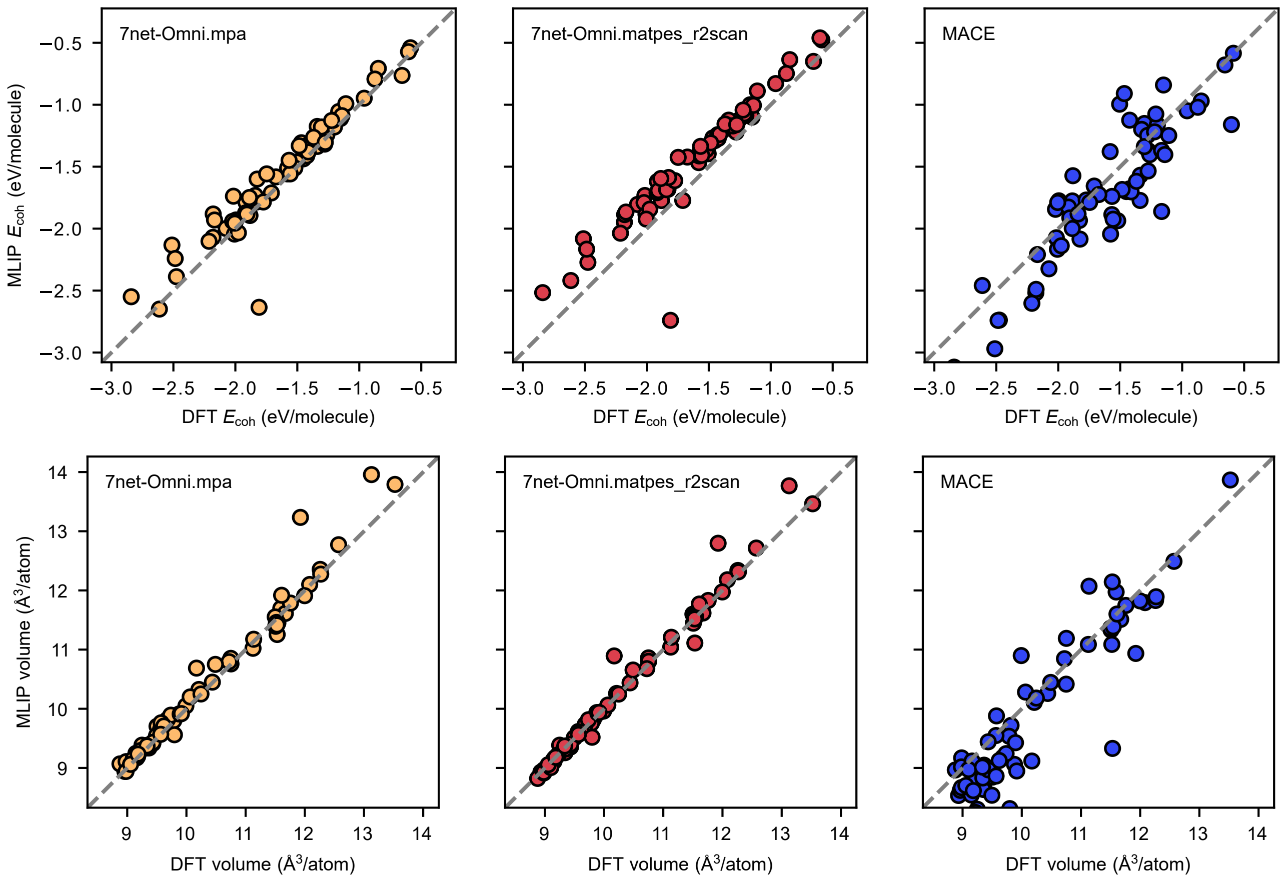

Supplementary Figure 31: **Parity plots of cohesive energies and equilibrium volumes in BMCOS1 benchmark.** Reference values are calculated at the r^{2}SCAN-D3 level of theory. The first row illustrates results on cohesive energies and the second row depicts plots of equilibrium volume of molecular crystals.

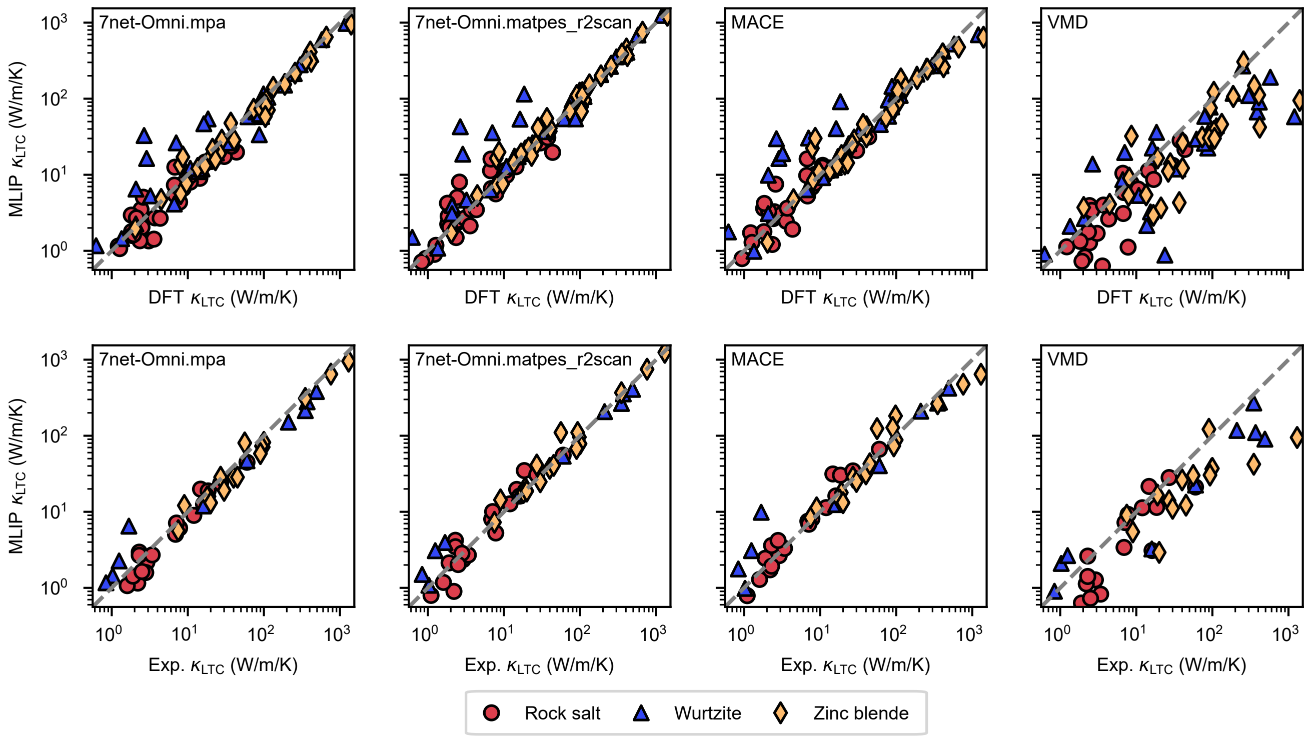

Supplementary Figure 32: **Parity plots of lattice thermal conductivity (κ_{LTC}) in binary solids.** The first row illustrates MLIP results compared to κ_{LTC} calculated by r^{2}SCAN functional while the second row depicts correlation compared with experimental κ_{LTC}. Circle, triangle and diamond markers indicate prototype of corresponding materials (rock salt, wurtzite and zinc blende, respectively).

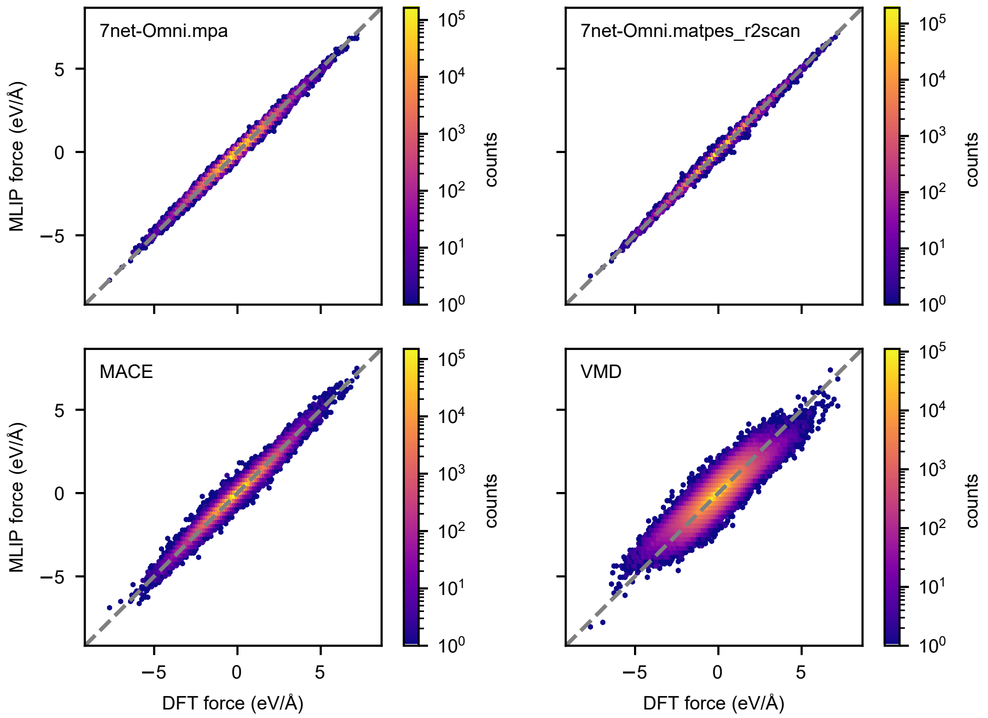

Supplementary Figure 33: **Parity plots of force components obtained from ab initio molecular dynamics simulations of argyrodite Li_6PS_5Cl.** Molecular dynamics simulations are conducted using r^{2}SCAN functional. Color at each region indicates the count of data points.

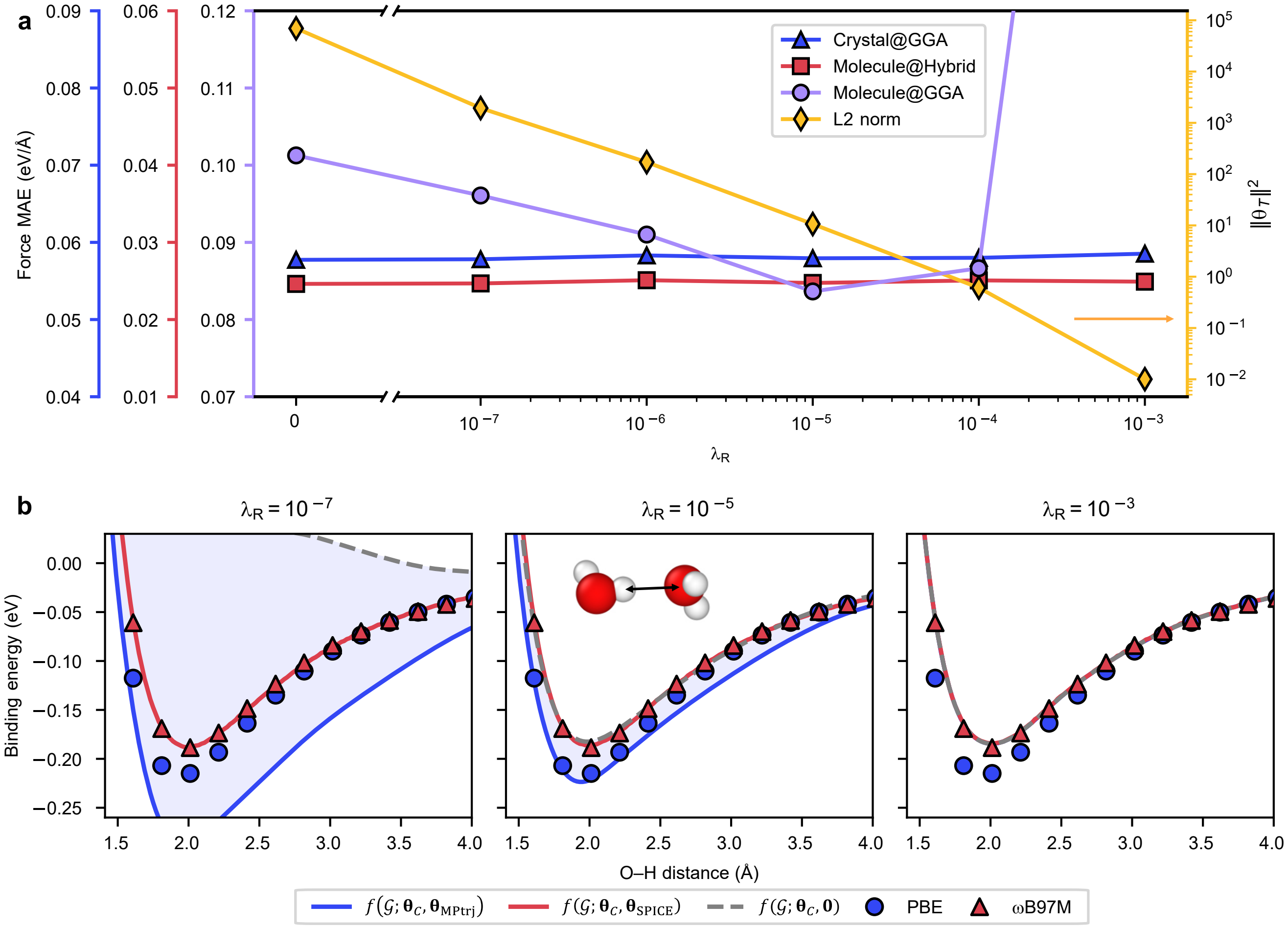

Supplementary Figure 34: **Performance of multi-task models by varying regularization loss weight (λ_R). a** Force MAE and L2 norm trend with selection of λ_R. Blue and purple plots show force MAE in MPtrj channel compared with the PBE reference for crystal and molecular structures, respectively. The red plot shows the force MAE in the SPICE channel compared with the ωB97M reference. The yellow plot shows the L2 norm of task-specific parameters decreases as λ_R increases. **b** Potential energy surface of water dimer binding. Blue and red lines show PES calculated with MPtrj channel and SPICE channel, respectively. Gray dashed lines show common PES contributions, obtained with inferring models by setting task-specific parameters to zeros. The blue shaded region shows the task-specific contribution for MPtrj channel. Blue and red markers indicate reference binding energy calculated with corresponding ab initio methods.

Supplementary references

[1] Larsen, A.H., Mortensen, J.J., Blomqvist, J., Castelli, I.E., Christensen, R., Dułak, M., Friis, J., Groves, M.N., Hammer, B., Hargus, C., Hermes, E.D., Jennings, P.C., Jensen, P.B., Kermode, J., Kitchin, J.R., Kolsbjerg, E.L., Kubal, J., Kaasbjerg, K., Lysgaard, S., Maronsson, J.B., Maxson, T., Olsen, T., Pastewka, L., Peterson, A., Rostgaard, C., Schiøtz, J., Schütt, O., Strange, M., Thygesen, K.S., Vegge, T., Vilhelmsen, L., Walter, M., Zeng, Z., Jacobsen, K.W.: The atomic simulation environment—a Python library for working with atoms. J. Phys.:Condens. Matter **29**(27), 273002 (2017)

[2] Cordero, B., Gómez, V., Platero-Prats, A.E., Revés, M., Echeverría, J., Cremades, E., Barragán, F., Alvarez, S.: Covalent radii revisited. Dalton Trans. (21), 2832–2838 (2008)

[3] Yohannes, A.G., Lee, C., Talebi, P., Mok, D.H., Karamad, M., Back, S., Siahrostami, S.: Combined High-Throughput DFT and ML Screening of Transition Metal Nitrides for Electrochemical CO_2 Reduction. ACS Catal. **13**(13), 9007–9017 (2023)

[4] Jain, A., Hautier, G., Ong, S.P., Moore, C.J., Fischer, C.C., Persson, K.A., Ceder, G.: Formation enthalpies by mixing GGA and GGA+U calculations. Phys. Rev. B **84**(4), 045115 (2011)
